# Transcriptome and proteome analyses reveal genes and signaling pathways involved in the response to two insect hormones in the insect-fungal pathogen *Hirsutella satumaensis*

**DOI:** 10.1128/msystems.00166-24

**Published:** 2024-07-10

**Authors:** Jiaojiao Qu, Yongli Feng, Xiao Zou, Yeming Zhou, Wei Cao

**Affiliations:** 1 College of Tea Sciences, Guizhou University, Guiyang, China; 2 Institute of Fungal Resources, College of Life Sciences, Guizhou University, Guiyang, China; 3 Key Laboratory of Plant Resource Conservation and Germplasm Innovation in Mountainous Region (Ministry of Education), College of Life Sciences/Institute of Agro-bioengineering, Guizhou University, Guiyang, China; University of Toronto, Toronto, Ontario, Canada

**Keywords:** *Hirsutella satumaensis*, ecdysone, juvenile hormone III, transcriptome and proteome, conidia mucilage

## Abstract

The insect hormones ecdysone (20E) and juvenile hormone III (JH) have been demonstrated to stimulate the secretion of conidia mucilage and pigments in *Hirsutella satumaensis*. However, the underlying mechanisms remain elusive. Here, comparative transcriptome and proteome analyses were performed to identify the fungal genes and proteins of *H. satumaensis* that are up- or downregulated in response to insect hormones. A total of 17,407 unigenes and 1,016 proteins in conidia mucilage were identified. The genes involved in response to the hormones were classified into four functional groups: (1) stress response-related genes that are required for the removal of reactive oxygen species (glutathione synthetase, c7144) and genes involved in the response to osmotic stress in the hemocoel, such as those encoding proteins involved in the G, mTOR, and MAPK signaling pathways (2); insect hormone metabolic genes, including genes encoding ecdysteroid UDP-glucosyltransferase, ecdysteroid-22-kinase, and a key aldehyde dehydrogenase in a juvenile hormone synthesis pathway (3); secretory proteins that share homology with those of the host *Bombyx mori*, including fibrohexamerin, sericin 1, metalloprotease 1 protein, and silk gum protein, which were revealed by the omics data; and (4) proteins related to amino sugar metabolism and oxidative phosphorylation that were specifically expressed in mucilage in response to 20E and JH, respectively. These findings revealed that *H. satumaensis* can mount effective responses by modulating the expression of genes involved in the detoxification, adaptation, and evasion of insect hormone-mediated immune responses, providing fresh insights into fungal pathogen-host insect interactions.

**IMPORTANCE:**

Insect hormones are highly important for the regulation of insect growth, development, and immune system function. Thus, the expansion of entomopathogenic fungi (EPF) could be affected by these hormones when they inhabit the host hemocoel. However, the molecular basis of EPF in response to insect hormones has yet to be determined. Our results revealed that EPF are impacted by 20E and JH, both of which act as signals, as these hormones lead to changes in metabolic pathways of the fungus, thus demonstrating a direct relationship between the fungus and the hormones. Furthermore, adaptive strategies, such as the use of ecdysone-inactivating enzymes and secreted filamentous proteins in *H. satumaensis*, which strongly resemble those of the host insect, have been discovered, thus illustrating the importance of adaptation to insect hormones for a better understanding of the interaction between insects and EPF.

## INTRODUCTION

Entomopathogenic fungi (EPF) are a class of fungi that can cause disease and death in insects. They are able to penetrate the cuticle of insects to enter the hemocoel. Due to their environmental friendliness, specificity, strong virulence, and persistent insecticidal effects, EPF are ideal for the development of fungal insecticides (1, 2). This infection mode, however, is accompanied by slow killing and their spores are easily affected by the field environment (3). To overcome these obstacles, it is essential to gain insight into the pathogenic mechanisms of each link between fungal infection and disease to detect the functional genes and signaling pathways associated with pathogenicity for genetic improvement. Much work has been done to investigate the colonization in the body cavity of insects by EPF, which has led to significant progress.

Upon entering the insect hemocoel, fungi are exposed to increased levels of reactive oxygen species (ROS), elevated osmotic pressure, specialized nutrients, and a variety of immune responses (4). To colonize the hemocoel of insects, EPF has developed a range of adaptive strategies. To evade the humoral and cellular immune responses of insects (including hemocytosis, endocytosis, and melanosis, antimicrobial peptides, and other fungal virulence factors), fungi can alter their cell morphology, such as forming a yeast-like spore or mycelium by modifying the cell wall structure and employing protective layer proteins to disguise the immunogenic carbohydrates present on the cell surface (5–7). Fungal cells can evade host recognition and rapidly escape from blood cells (8, 9). Following penetration, the fungus adapts to the conditions in the hemocoel by turning off the production of serine proteases, thereby preventing the activation of the prophenoloxidase system, which is related to melanin synthesis in insect cells (10). Moreover, a series of secondary metabolites with insecticidal activity are secreted, causing the host to resist feeding and weakening the insect’s immune response (11).

In addition to the stress factors mentioned above, insect hormones are also important factors in fungal proliferation, as they are key regulators of insect growth and contribute to regulating the fungal immune system. For example, ecdysone (20E) has been identified as one of the most essential hormones for the growth and development of insects (12–14). It has a crucial influence on humoral and cellular immunity and is responsible for the commencement of insect immune system development at an early stage (15). Numerous studies have revealed that 20E has a positive influence on the immune response of insects (16, 17). 20E binds to its receptor complex, LmEcR, and further activates the *LMIpl-1* gene, which controls the antifungal cells and humoral immune response of migratory locusts. After silencing *LMIPL-1*, the plasma of migratory locusts showed a significant decrease in antifungal activity, whereas the mortality of insects treated with spores of *Metarhizium acridum* was significantly greater than that of the control group (18). Recent studies have indicated that 20E can modulate the expression of the Toll-like receptor (*TLR*), a key gene involved in locust immunity, through its association with the ecdysone receptor (*LmEcR*). *LmTLR* is expressed in the epidermis, and both internal and external sources of 20E can stimulate its expression (19). It is, therefore, conceivable that 20E may impede the growth of EPF in the insect hemocoel and plays an important role in mediating responses to fungal infection. Moreover, some insect pathogenic fungi have evolved mechanisms for targeting this hormone as a means of facilitating infection (20).

There is an ongoing debate concerning the correlation between juvenile hormone III (JH) and immune regulation in insects. Research conducted on *Tenebrio molitor* revealed that JH has a detrimental effect on insect immunity, including the suppression of the phenoloxidase (PO) cascade and hemocyte encapsulation (21). Other studies have demonstrated that JH plays a role in regulating the immune response in male *Aedes aegypti* after emergence but has a suppressive effect in females after emergence (22, 23). Experiments conducted by Rantala et al. (23) revealed that JH administration prolongs the survival time of male mealworm beetles and *Tenebrio molitor* after infection with *M. robertsii* but reduces survival time in females. Despite the need for further investigation into the regulation of insect immunity by JH, it is evident that 20E and JH can regulate the immune system of insects, thus influencing the colonization of the host by EPF. Nevertheless, the precise effects of insect hormones on pathogenic fungi remain limited.

*Hirsutella* is a globally distributed genus of insect fungal pathogens that is obligatorily parasitic to arthropods and nematodes (24) and is an asexual stage of Ophiocordycipitaceae fungi. In comparison to the broad-host-range EPF, such as *Beauveria* and *Metarhizium*, the majority of *Hirsutella* species are known to have a more specific host range. The spores are usually larger and fewer in number, and their surfaces are typically covered with a thick mucilage layer, which facilitates adhesion and recognition on the host surface (25). *H. satumaensis* (26) is a species that possesses unique characteristics within this genus. It is an obligate parasite of Lepidopteran insects with a relatively broad host range, rapid growth rate, and prolific spore production (27). Despite the differences in growth rate and sporulation rate from other broad host range EPF such as *B. bassiana*, this fungus can still cause host population epidemics in the natural environment, having its particularities in terms of environmental adaptability and host infection mechanism (28).

Our previous study showed that the secretion of pigments and conidial production in *H. satumaensis* were altered after incubation with 20E and JH (29). With increasing hormone concentrations, the pigment circle and mucilage thickness of the conidia increased (Fig S1. and S2), while sporulation initially increased and then decreased slightly, indicating that the two insect hormones might influence the growth of the fungus during the colonization process in insects (30). These findings indicate that *H. satumaensis* may be directly influenced by these two hormones during the invasion of the hemocoel region of insects. While the fungus is believed to directly engage with insect hormones, the scope and mechanism of the fungal response remain unclear. This investigation leveraged comparative transcriptomics and proteomics analysis to explore the molecular underpinnings of *H. satumaensis* in response to these two insect hormones.

## RESULTS

### Transcriptional response of *H. satumaensis* to insect hormones

As the *H. satumaensis* genome is unavailable, the RNA sequencing (RNA-seq) data were *de novo* assembled using Trinity. These clean reads were assembled into 31,759 transcripts and subsequently assembled into 17,407 unigenes with an average length of 1,452 bp and an N50 of 4,377 bp. Among the sequenced fragments, the shortest transcript was 201 bp and the longest transcript was 23,001 bp. We further determined the distribution of the lengths of these fragments. With 500 bp as the statistical unit, approximately 70% of the sequenced fragments were distributed between 201 and 1,500 bp (Fig. S3A). BUSCO software was used to assess the coherence of the assembly outcomes, and 96.5% of the complete single-copy genes were assembled as detailed in Table S1. An analysis was conducted on the *E*-value distribution, similarity distribution, and species distribution of unigenes from *H. satumaensis* that were matched to the NR (non-redundant protein sequence) database. According to the *E-*value distribution, 70.86% of the unigenes exhibited high homology (<1e^−10^) (Fig. S3B). The similarity distribution of the unigenes revealed that the majority (90.13%) had a similarity of at least 60% (Fig. S3C). For the species distribution, the annotated *H. satumaensis* unigenes showed the greatest similarity to *Ophiocordyceps sinensis* CO18, with 2,635 matching genes (23.35%), illustrating the close relationship between these two species (Fig. S3D).

To gain insight into the correlation between the samples, particularly the biological replicates, and assess the validity of the experimental design, we conducted a correlation analysis on nine samples (Fig. S4). Principal component analysis revealed that the biological repeat samples from the same treatment group formed an independent cluster, indicating a high degree of repeatability. The PCA diagram revealed a distinct separation between the three groups of samples, demonstrating that the results of insect hormone treatment were clearly different from those of the control group. The process of functional annotation was performed by searching all sequenced *H. satumaensis* unigenes against public databases. As a result, 14,911 unigenes (75.7%) were successfully annotated and functionally classified in the Swiss-Prot (12,516), NR (10,978), KEGG (9,106), GO (4,985), and Clusters of Orthologous Genes (COG) (3,481) databases. The details of this annotation and classification can be found in Table S2.

**Fig 1:**
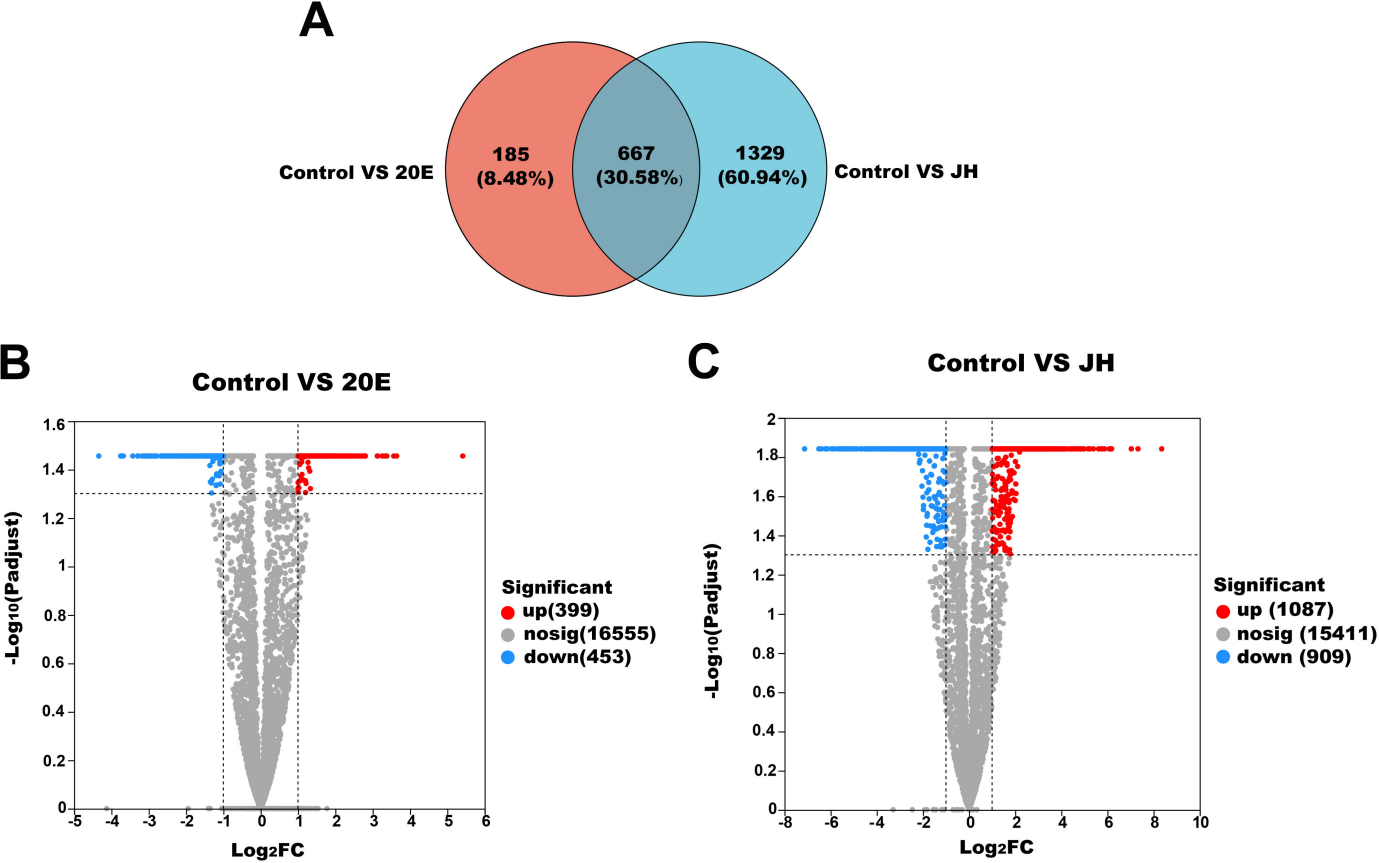
Differentially expressed genes (DEGs) analysis. (A) Number of upregulated and downregulated DEGs associated with the two insect hormones under the induced status in *H. satumaensis* (20E: ecdysone, JH: juvenile hormone III; same below). (B and C) Volcanograms of DEGs under 20E and JH pressure, respectively. (D and E) KEGG enrichment analysis of significantly up and downregulated unigenes of *H. satumaensis* under 20E and JH stress, respectively.

Compared with those in the control, a total of 185 unigenes (8.48%) and 1,329 unigenes (60.94%) exhibited significant changes in expression when the fungus was exposed to 20E and JH, respectively, while 667 unigenes (30.58%) showed similar expression levels under both treatments (Fig. 1A). A total of 852 unigenes were significantly altered under 20E stress, including 399 upregulated and 453 downregulated unigenes (Fig. 1B; Table 1). JH stress caused a significant alteration in 1,996 unigenes, comprising 1,087 upregulated and 909 downregulated unigenes (Fig. 1C; Table 1).

**TABLE 1:** Distribution of significantly differentially expressed genes from *H. satumaensis*

Stress	Total	Number of genes expressed in fold change					
		Upregulated			Downregulated		
		>2	>4	>8	>2	>4	>8
JH	1,996	1,087	371	153	909	779	112
20E	852	399	155	39	453	203	38

**Fig 2:**
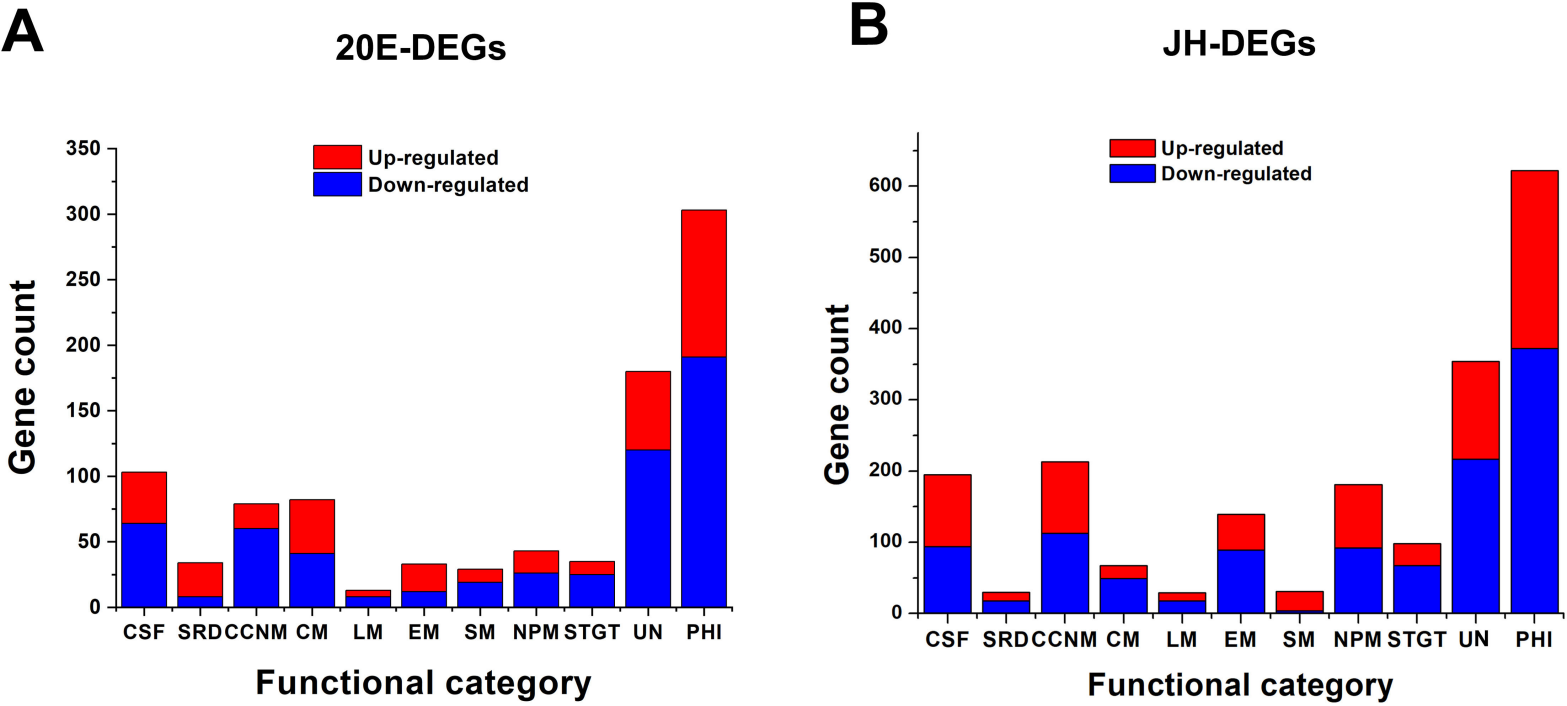
The functional classification of significantly up- and downregulated DEGs of *H. satumaensis* under the stress of 20E and JH, respectively. The abbreviations for the functional classification are CSF (cell structure and function), SRD (stress response and defense), CCNM (cell cycle and nucleic acid metabolism), CM (carbohydrate metabolism), LM (lipid metabolism), EN (energy metabolism), SM (secondary metabolism), NPM (nitrogen and protein metabolism), SITG (signal transduction and gene transcription), UN (unknown), and PHI (pathogen-host interactions).

To gain insight into the significant upregulation and downregulation of genes in *H. satumaensis* exposed to 20E and JH, a sequence query and function search was conducted using GO term enrichment analysis. In the 20E and JH treatment groups, a total of 29 GO categories were assigned to both the up- and downregulated differentially expressed genes (DEGs) (Fig. S5). GO term enrichment analysis categorized the annotated sequences into three main categories, biological process, cellular component, and molecular function, in both the 20E and JH treatment groups. The biological process category was mainly composed of “metabolic process,” followed by “cellular process” and “single-organism process.” In the cellular component category, “cell” and “cell part” were the dominant categories, followed by “organelle.” In terms of molecular function category, “binding” was the most dominant group, followed by “catalytic activity.” To gain a better understanding of the role of DEGs in *H. satumaensis* under the influence of these two hormones, we classified these genes into 11 functional categories, namely, cell structure and function, stress response and defense, cell cycle and nucleic acid metabolism, carbohydrate metabolism, lipid metabolism, energy metabolism, secondary metabolism, nitrogen and protein metabolism, signal transduction and gene transcription, unknown, and pathogen-host interactions (Fig. 2A and B).

Pathogen-host interaction (PHI) and unknown DEGs were the top two terms for both the 20E and JH treatments. In the 20E treatment group, most DEGs were classified in the PHI category, in which 112 DEGs were significantly upregulated with log2 ratios ranging from 1.0 to 5.42, and 191 were downregulated, with log2 ratios ranging from −4.33 to −1.01. These DEGs included genes potentially associated with pathogen-host interactions and virulence factors, including chitinase, killer toxin, concanavalin A-like lectin, metalloprotease 1 protein, pheromone receptor, secreted protein, and transporters (Fig. 2A, Fig. S5A, Table S3). The second term was enriched for functionally unknown genes, which could be linked to the incomplete genomic information of *H. satumaensis*. Moreover, a minimum twofold difference in expression was observed for 39 upregulated and 64 downregulated unigenes related to cell structure and function, with log2 ratios ranging from 1.0 to 3.57 and from −3.29 to −1.01, respectively.

Moreover, the majority of DEGs in the JH treatment group were related to the PHI. There were 250 upregulated DEGs with log2 ratios ranging from 1.01 to 6.14 and 372 downregulated DEGs with log2 ratios ranging from −6.51 to −1.01. Specifically, these DEGs included silk gum protein, stress-associated endoplasmic reticulum protein 2, and calcium-transporting ATPase 3 genes, which were different from those in the 20E treatment group. In the second item of the JH treatment group, 137 upregulated and 217 downregulated unigenes were classified as unknown, with log2 ratios ranging from 1.0 to 3.57 and −7.13 to −1.02, respectively. Additionally, the number of DEGs was greater in the cell structure and function (up to 101, with log2 ratios ranging from 1.01 to 3.39; down to 94, with log2 ratios ranging from −5.18 to −1.0), cell cycle and nucleic acid metabolism (up to 100, with log2 ratios ranging from 1.02 to 6.14; down to 92, with log2 ratios ranging from −6.51 to −1.0), and nitrogen and protein metabolism (up to 89, with log2 ratios ranging from 1.02 to 8.35; down to 94, with log2 ratios ranging from −6.14 to −1.03) categories than in the other categories. Thirty DEGs related to stress response and defense, such as the pH-responsive transcription factor pacC, heat shock protein 90, heat-labile enterotoxin, WSC domain-containing protein, DNA polymerase zeta catalytic subunit, and aerolysin-like toxin, were identified (Fig. 2B; Fig. S5B; Table S4). These findings suggest that fungal colonization of the insect hemocoel is likely affected by these two insect hormones, as indicated by the differential expression of genes related to host interactions.

The COG classifications of the DEGs in the two insect hormone treatment groups are shown in Table S5. The DEGs from both the 20E and JH groups were divided into 25 categories based on their COG functional properties. The category of function unknown (S) was the most prevalent among both groups of DEGs. In the 20E-induced group, the largest COG category was associated with amino acid transport and metabolism (E, 107), lipid transport and metabolism (I, 75), and carbohydrate transport and metabolism (G, 60). There were more DEGs related to translation, ribosomal structure and biogenesis (J, 321), amino acid transport and metabolism (E, 158), and general function prediction only (R, 127) in the JH-induced group.

KEGG analysis indicated that the DEGs in both groups could be classified into 22 pathways. In the 20E treatment group, the upregulated DEGs associated with ribosome, oxidative phosphorylation, and the biosynthesis of amino acids were significantly enriched in four metabolic pathways—tyrosine (map 00350), arginine and proline (map 00330), arginine biosynthesis (map 00220), and phenylalanine metabolism (map 00360) (Fig. S6A), while the downregulated DEGs were significantly enriched in lysine biosynthesis (map 00300) and aflatoxin biosynthesis (map 00254) (Fig. S6B). These results suggest that the DEGs associated with the response to 20E were involved mainly in the synthesis and metabolism of amino acids. In the JH group, DEGs were found to be significantly upregulated in the ribosome metabolic pathway (map 03010), while those DEGs were enriched in the pathways of indole diterpene alkaloid biosynthesis (map 00403), ubiquinone and other terpene-quinone biosynthesis (map 00130), and arachidonic acid metabolism (map 00590) (Fig. S7).

### Metabolic pathways of *H. satumaensis* in response to the two insect hormones

To further understand the secondary biosynthetic and regulatory pathways modulated by the two hormones, iPath 2.0 was used to visualize the metabolic pathways of those DEGs. The two DEG pools shared common annotated pathways, such as nucleic acid metabolism, carbon metabolism, amino acid metabolism, and energy metabolism (Fig. S8). The pathways of steroid and diterpenoid biosynthesis were specifically enriched for 20E-induced DEGs, as depicted in Fig. S8. The regulatory pathways of the DEGs specifically induced by JH were enriched in insect hormone biosynthesis (depicted by black circles in Fig. S8) and the processes of protein translation, folding, sequencing, and secretion in the ribosome. These metabolic pathways seem to directly target insect hormones, such as proteins involved in transcription and translation, and some enzymes are involved in the metabolism of ecdysone and juvenile hormone III.

In the steroid biosynthetic pathway of the 20E-induced group, the upregulated DEGs were related to ecdysone metabolism (c10187, c6594, and c8461). An aldehyde dehydrogenase gene (c10061), which is markedly upregulated in the insect hormone biosynthesis pathway, was notably enriched after JH induction (Table 2).

**TABLE 2:** Genes related to hormone metabolism in DEGs of hormone treatment group

Gene ID	Group	Log2FC ratio	Swiss-Prot description
c10187	20E	2.89	Ecdysteroid UDP-glucosyltransferase
c841	20E	1.89	Ecdysteroid kinase
c6594	20E	1.25	Protein kinase-like domain
c10061	JH	3.21	Aldehyde dehydrogenase

**Fig 3:**
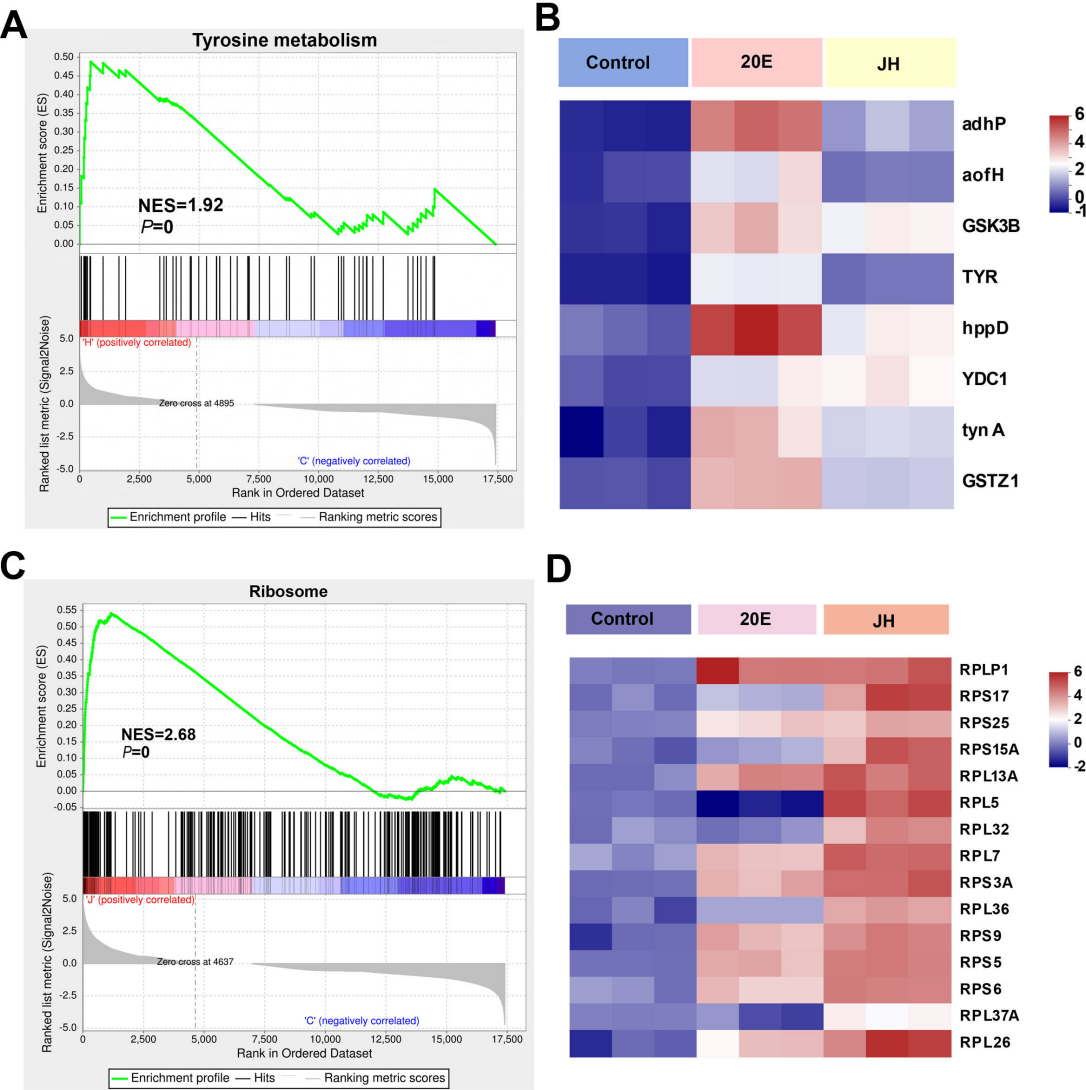
The enrichment score of the metabolic pathway and the top genes expressed in the 20E and JH groups were analyzed by GSEA based on the RNA-seq data sets. The 20E treatment group showed significant enrichment in the tyrosine pathway (**A**), while the ribosome pathway was notably enriched in the JH treatment group (**C**). The top 8 and 15 most highly expressed genes in the 20E (**B**) and JH (**D**) groups, respectively. NES, a normalized enrichment score. *P* values in the graphs were calculated by GSEA analysis.

To further reveal the signaling pathways associated with the phenotypes of *H. satumaensis*, gene set enrichment analysis (GSEA) was performed. The DEGs in the 20E group were mainly linked to tyrosine metabolism, ribosome metabolism, protein processing in the endoplasmic reticulum, RNA transport, and the MAPK signaling pathway (Fig. S9A), while the DEGs in the JH group were mainly associated with the ribosome pathway, MAPK signaling pathway, glycolysis/gluconeogenesis, and amino sugar and nucleotide sugar metabolism (Fig. S9B). These results were consistent with the KEGG pathway analysis, suggesting that tyrosine metabolism and the ribosome pathway might be significantly regulated in response to 20E and JH, respectively (Fig. 3A and C). In the tyrosine metabolism pathway, eight of the top genes, such as *ADHP* (alcohol dehydrogenase), *GSTZ1* (glutathione S-transferase), and *HPPD* (4-hydroxyphenylpyruvate dioxygenase), were significantly upregulated in response to 20E (Fig. 3B).

Furthermore, the ribosome pathway was notably enriched in both treatment groups (Fig. S9). We compared the DEGs in this pathway between the two treatment groups and revealed that 71 genes were co-expressed, 16 genes were specifically expressed in the JH treatment group, and 3 genes were expressed in the JH treatment group. The expression of the top 15 co-expressed genes that differed between the 2 groups were related to protein production and regulation, such as *RPL5*, *RPL32*, and *RPS15A*, which were highly expressed in the JH group but downregulated in the 20E group (Fig. 3D).

### Secretory proteins of fungal pathogens in response to the two hormones

After exposure to the two insect hormones, the colony of *H. satumaensis* displayed a significantly enlarged pigmentation circle and a distinctly thickened conidial mucilage (Fig. S1 and S2). To further reveal the underlying mechanism, extracellular secretory proteins encoded by DEGs were identified compared to controls (without induction). Proteins with N-terminal signal peptides but without transmembrane structures or other organelle localization signals are likely to be either cell surface or secreted proteins. The expression of 26 secreted protein genes encoding serine protease, killer toxin, metalloprotease, secreted protein, and chitinase was upregulated in the 20E treatment group (Table S6-1). A total of 51 upregulated secreted protein genes encoding fibrohexamerin, sericin 1, metalloprotease 1 protein, silk gum protein, serine protease lipase, chitinase, and other secreted silk proteins were upregulated in the JH group (Table S6-2).

**Fig 4:**
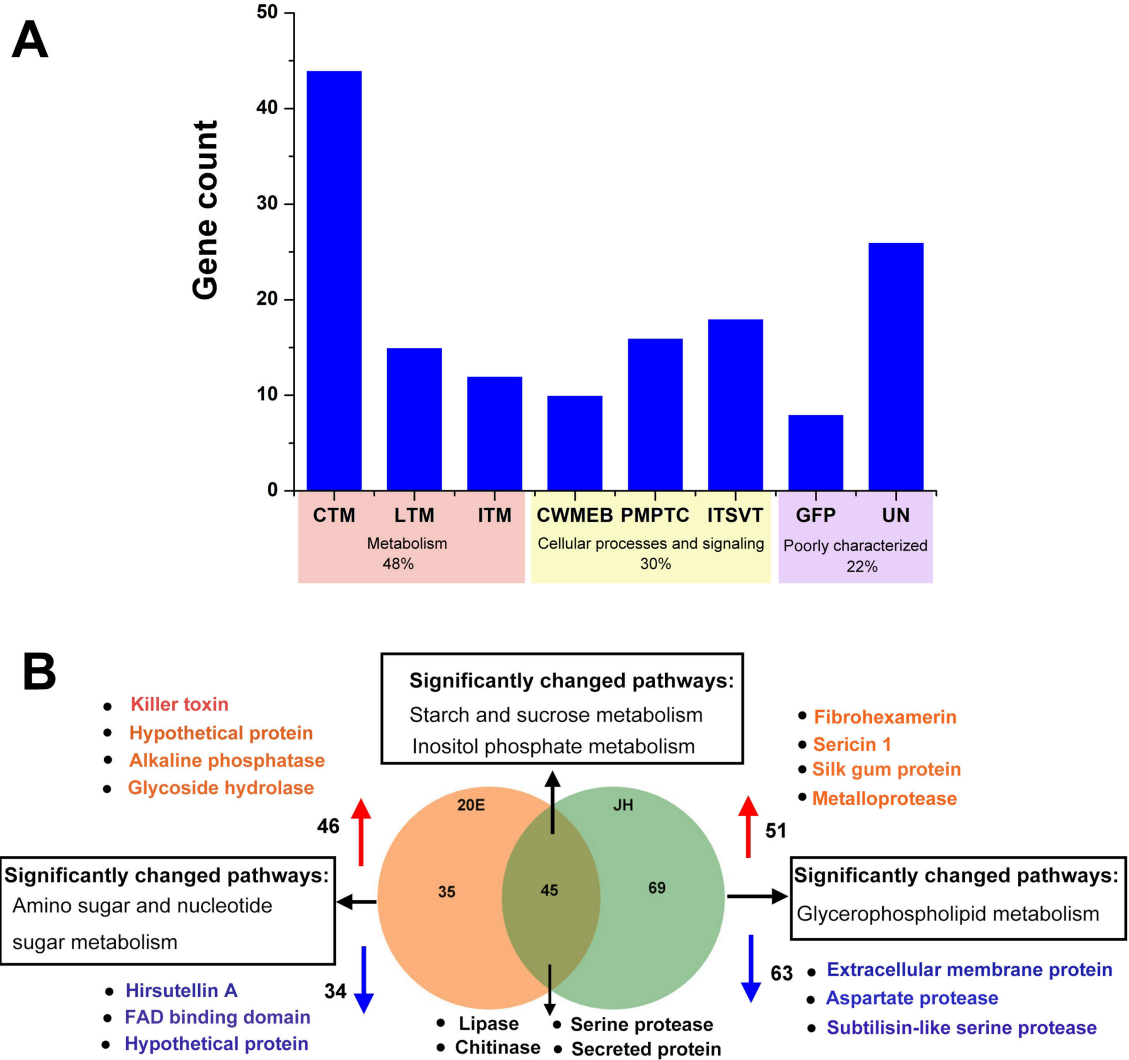
Classification and analysis of secretory proteins from the transcriptome data of *H. satumaensis* under 20E and JH induction conditions. (A) Functional classification of the co-expressed secretory proteins. (B) The Venn diagram shows the number of identified proteins that are significantly up- and downregulated. The functional classification abbreviations are CTM (carbohydrate transport and metabolism), LTM (lipid transport and metabolism), ITM (inorganic ion transport and metabolism), CWMEB (cell wall/membrane/envelope biogenesis), PMPTC (post-translational modification, protein turnover, chaperones), ITSVT (intracellular trafficking, secretion and vesicular transport), GFP (general function prediction only), and UN (unknown).

A functional classification of the secreted proteins co-expressed in the two hormone-induced groups revealed that 48% of the secreted proteins were linked to secondary metabolism, and other proteins were involved in the stress response, cell rescue, and detoxification. Approximately 30% of the proteins in the functional annotation are associated with nutrition and energy metabolism, including carbohydrate, nitrogen, nucleic acid, and energy metabolism, while the remaining genes remain unidentified (Fig. 4A). Moreover, a Venn diagram indicated that the two treatment groups exhibited the co-expression of 45 protein genes, encoding lipases, chitinases, serine proteases, and some secreted proteins with unknown roles. The two main metabolic pathways for the enrichment of these proteins were starch and sucrose metabolism and inositol phosphate metabolism (Fig. 4B). In the 20E group, most of the 35 secreted proteins were associated with amino sugar and nucleic acid metabolism. Notably, the expression of hirsutellin A, a ribotoxin exclusive to *Hirsutella*, decreased. Sixty-nine secreted proteins exclusive to the JH treatment group were significantly enriched in the glycerophospholipid metabolism pathway. These proteins, such as sericin1, fibrohexamerin, and silk gum protein, were highly homologous to those of the host *Bombyx mori*, and their gene expression was significantly upregulated.

The hierarchical clustering analysis revealed that DEGs encoding secreted proteins were upregulated to different extents in both treatment groups (Fig. S10A), which might result in morphological changes as the pigment of the colony and the mucilage of the conidia increased (Fig. S1 and S2). Moreover, the genes encoding secreted proteins were divided into four clusters. The genes in Clusters 2 and 4 were upregulated in response to the two insect hormones involved in the glycerolipid metabolism (map00561) and folate biosynthesis (map00790) pathways. However, the genes in Cluster 1 were downregulated, while those in Cluster 3 were significantly upregulated in response to 20E. Both clusters were significantly enriched in the amino sugar and nucleotide sugar metabolism (map00520) pathway (Fig. S10B).

### Specifically expressed proteins in conidia extracellular mucilage in response to 20E and JH

**Fig 5:**
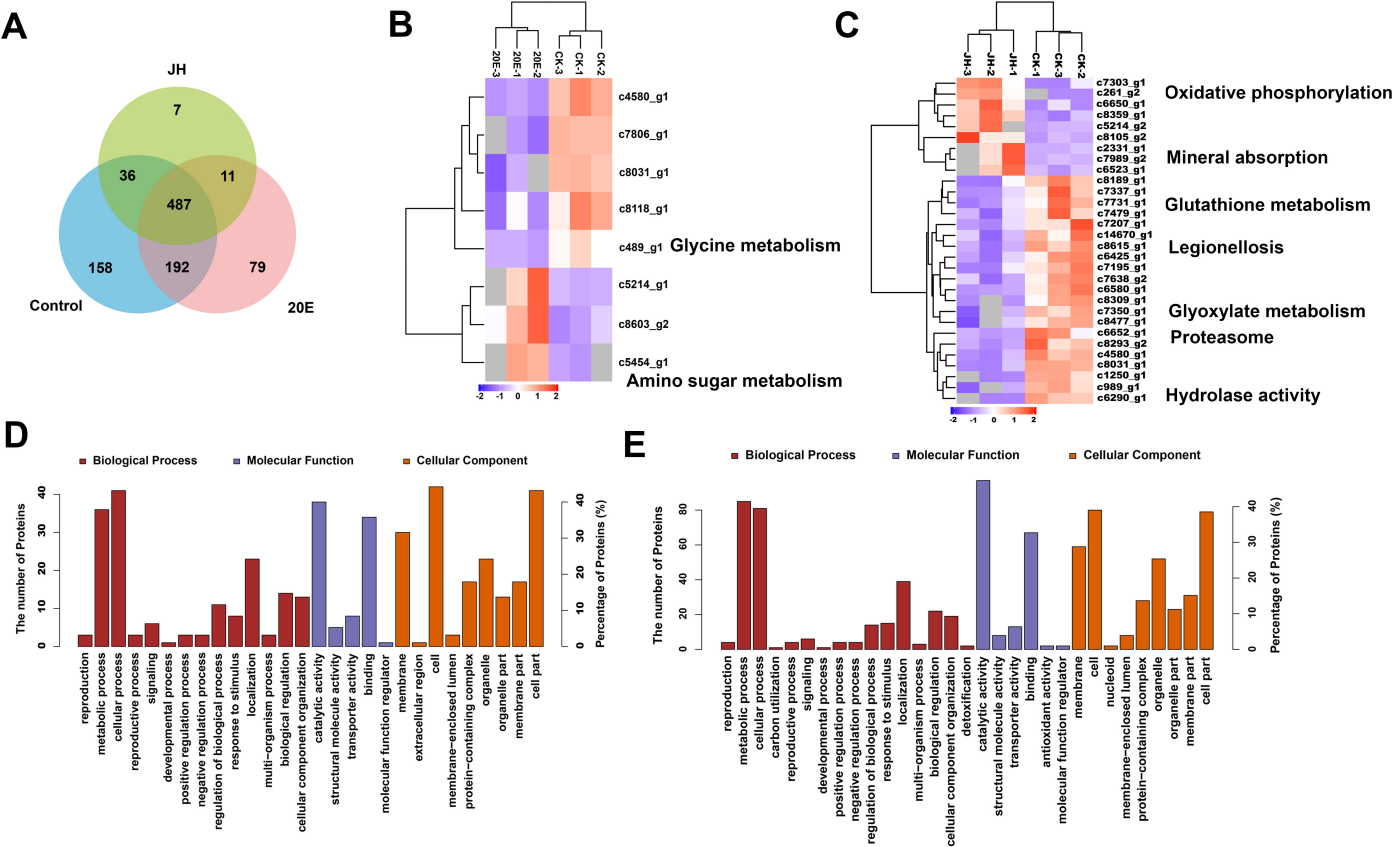
Analysis of differentially expressed proteins (DEPs). (A) Number of DEPs associated with mucilage induced by the two insect hormones. (B and C) Hierarchical clustering of specifically expressed proteins in the 20E and JH treatment groups, respectively. (D and E) GO classes of DEPs associated with the mucilage of *H. satumaensis* under 20E and JH induced, respectively.

The two insect hormones have a notable effect on the extracellular mucilage thickness of *H. satumaensis*. The transcriptome data revealed that a set of genes encoding secretory proteins were significantly upregulated in response to the two hormones. These results suggest that the alteration in mucilage thickness on the conidia may be linked to an increased production of secretory proteins. To confirm this linkage, label-free protein quantification methods were employed to identify the extracellular mucilage proteins of *H. satumaensis* spores in response to 20E and JH compared to those of the control spores (without induction). A total of 1,016 proteins were identified, with 827, 726, and 504 proteins identified in the control, 20E and JH treatment groups, respectively. The majority of the proteins had a relative molecular mass between 10 and 85 kDa (Fig. S11). Changes in abundance at the protein level were considered significant if they satisfied a twofold cutoff relative to the control at a false discovery rate of less than 0.05. The 20E treatment group had 46 upregulated and 57 downregulated proteins, while the JH treatment group had 78 upregulated and 146 downregulated proteins (Table S7-1). The three samples contained a total of 487 proteins, 79 of which were exclusively identified in the 20E treatment group and 7 in the JH treatment group (Fig. 5A).

We subsequently performed a cluster analysis to identify the expression trends of the DEGs encoding secreted proteins in the three samples. One hundred nineteen proteins were present in all three groups and could be categorized into six clusters (Fig. S12). Cluster 1 had the greatest number of proteins (31), in which the protein abundances in the control group were greater than those in the 20E and JH groups. Clusters 2, 4, and 6 showed increased abundance in both hormone-treated groups, while the proteins of Cluster 3 were specifically upregulated in the JH group (Fig. S12). In the 20E treatment group, the specific upregulated proteins were clustered in the c5454 (amino sugar metabolism) gene, while the downregulated proteins were clustered in the c489 (glycine metabolism) gene (Fig. 5B). The JH treatment group displayed an increase in proteins annotated to the c261 and c6650 (oxidative phosphorylation) gene classes and a decrease in proteins annotated to the c7350 (glyoxylate metabolism) class (Fig. 5C).

To analyze the functions of proteins that were exclusively expressed in response to the hormones, GO annotation was performed (Fig. 5E and F). There were considerable similarities in the roles of spore mucilage-specific proteins between the two groups. For example, both groups were involved in energy metabolism, cell localization, and cell membrane localization. These results suggest that mucilage proteins of *H. satumaensis* might be essential for responding to different hormones and adapting to the insect environment. KEGG pathway enrichment analysis of these proteins with varying abundance levels was performed by Fisher’s exact test (Fig. S13). The proteins whose abundance significantly changed in the top 3 of the 20E treatment group were associated with amino sugar and nucleotide sugar metabolism (ko00520), glycine, serine, and threonine metabolism (ko00260), and the MAPK signaling pathway-yeast (ko04011) (Fig. S13A). The JH group was enriched in glutathione metabolism (ko00480); phenylalanine, tyrosine, and tryptophan biosynthesis (ko00400); and glycine, serine, and threonine metabolism (ko00260). The proteomic and transcriptomic data showed consistent results, confirming that the extracellular mucilage proteins produced by *H. satumaensis* were involved in the initiation of the host infection mechanism, amino carbohydrate metabolism, and cellular sensing in response to 20E and JH (Fig. S13B).

### Proteomic and transcriptomic data were combined to reveal the mechanisms underlying the response to insect hormones

**Fig 6:**
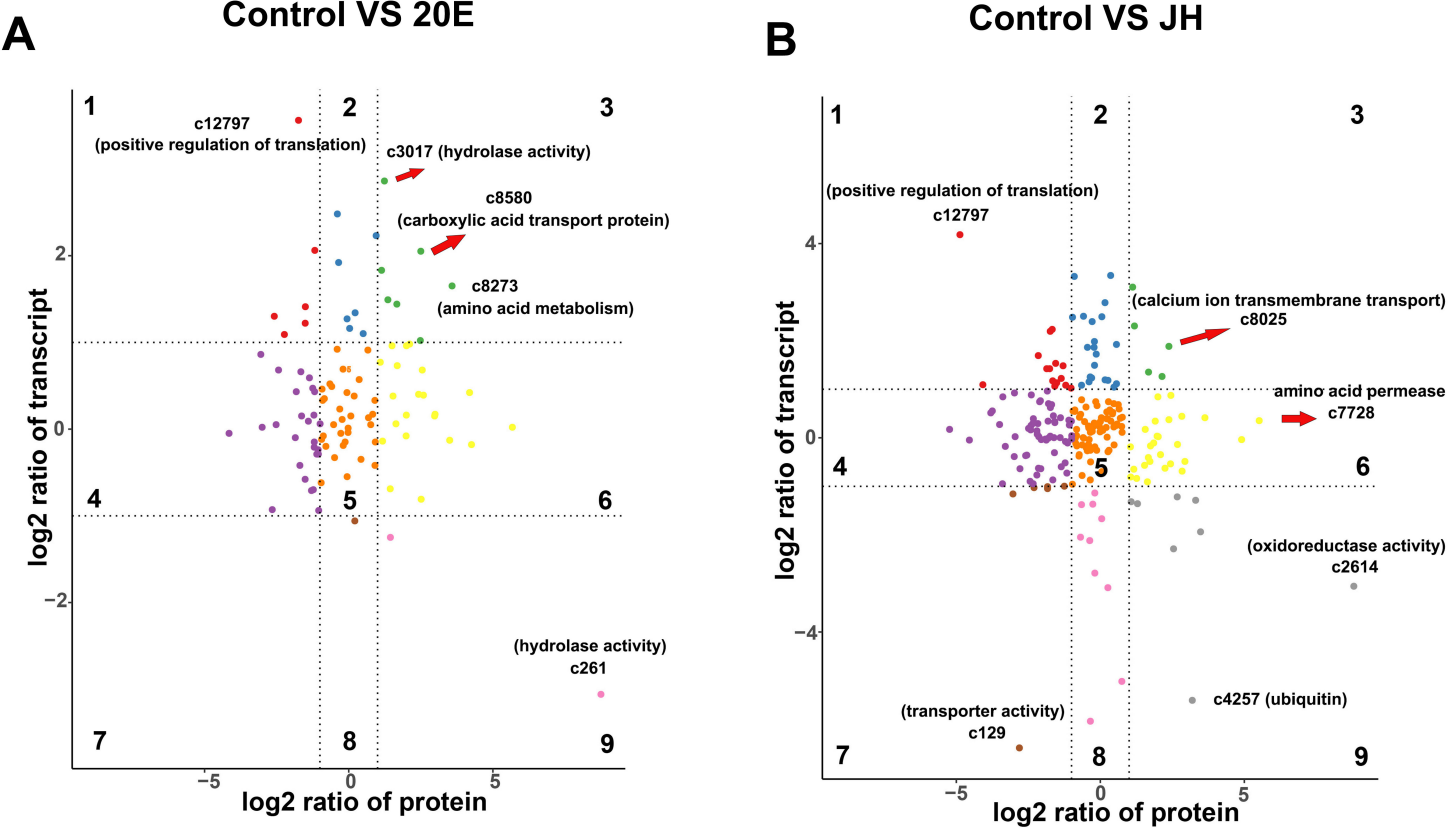
Candidates were divided into nine groups according to the log2 ratios of the protein species (*x*-axis) and transcripts (*y*-axis). Quadrants 1, 2, and 4 indicate that the protein abundance was lower than the RNA abundance. In quadrants 3 and 7, the RNAs correspond with the related proteins. Quadrant 5 shows that the proteins and RNAs were commonly expressed with no difference. Quadrants 6, 8, and 9 indicate that the protein abundance was higher than the RNA abundance. Comparison of changes in mRNAs and protein abundance of conidial mucilage.

The combined examination of transcriptomic and proteomic data offers a comprehensive approach, enhancing our understanding of the connection between the pathogenic fungus and two host hormones. Since the mucilage proteome data were obtained from the same conidial samples used to generate the transcriptome data, we performed a correlation analysis of the transcriptomes and proteomes. Scatterplot analysis of the log2-transformed ratios was used to show the distribution of the corresponding mRNA: protein ratios. The candidates detected in the proteome and transcriptome were divided into nine modules according to their expression patterns, establishing a nine-quadrant map (Fig. 6). A total of 1,681 mRNAs and 3,620 mRNAs changed in abundance in response to 20E and JH respectively, while 103 proteins and 224 proteins in the mucilage did so (Table 3). These values were associated with 28.7% and 61.9% of the evaluated transcripts and 14.2% and 41.4% of the evaluated mucilage proteins, respectively. In the nine quadrants analysis, in quadrants 1, 2, and 4, the protein abundance was lower than the RNA abundance. The protein abundance was greater than the RNA abundance in quadrants 6, 8, and 9. Quadrant 5 shows that the proteins and RNAs were commonly expressed with no differences. Quadrants 6, 8, and 9 indicated that the protein abundance was greater than the RNA abundance. After 20E treatment, 7 genes exhibited consistent expression of both mRNAs and corresponding proteins, while after JH treatment, 10 genes exhibited consistent expression (Table 3). Interestingly, we found that the number of genes in quadrants 1, 2, and 4 was greater than that in quadrants 6, 8, and 9 in both treatment groups, indicating that the majority of genes showed significant changes in mRNA levels, but not in protein abundance (Fig. 6). These results suggested that genes were regulated at the posttranscriptional or translational level when the fungus was in response to the two insect hormones, such as miRNA and lncRNA-mediated protein degradation.

**TABLE 3:** Combining transcriptomics and proteomics data sets based on the number of regulated mRNAs/proteins

	20E		JH	
	Transcriptomics	Proteomics	Transcriptomics	Proteomics
Evaluated mRNAs/proteins	5,849	726	5,849	541
Insect hormone regulated	1,681*^a^*	103^*b*^	3,620^*a*^	224^*b*^
Upregulated	762 (45%)	46 (45%)	1,723 (48%)	78 (35%)
Downregulated	919 (55%)	57 (55%)	1,897 (52%)	146(65%)
mRNA/protein expression trend coupled	7		10	

^*a*^ *P* < 0.01, and at least twofold regulation.

^*b*^ False discovery rate, 0.05, and at least twofold regulation.

Twenty-five candidates displayed significant changes in gene expression and protein abundance in both the transcriptome and proteome in the 20E groups, among which 12 and 13 candidates were upregulated and downregulated, respectively (Table S7-2). These proteins are involved in a variety of biological functions, such as cell wall processes, components or complexes of the cell membrane, transmembrane transport of molecules/ions, stress and resistance, oxidation-reduction processes, and carbon and energy metabolism (Fig. S13). Many of these genes are associated with pathogen-host interactions (PHI), suggesting that the response of *H. satumaensis* to insect hormones is likely an integral part of the fungal infection process. For example, c7951, a concanavalin A-like lectin/glucanase, was highly upregulated in both omics data sets. This lectin protein is likely to be distributed on the cell surface, suggesting that it plays an important role in immune recognition when the fungus encounters insect hormones.

A total of 30 candidates whose expression changed significantly were identified from the transcriptome and mucinous protein profiles in the JH treatment group. This group comprised 10 upregulated and 20 downregulated proteins (Table S7-3), which were involved in a variety of functions, such as membrane proteins, extracellular proteins, positive regulation of peptidase activity, biosynthesis of various amino acids, metal ion binding and catalytic activity, transmembrane transport activity, nucleic acid binding, fatty acid metabolism, and ATP binding. Two proteins were involved in the metabolism of filamentous proteins in the mycelial transcriptome and mucilage proteome. The genes c1056 and c15294 were identified in both profiles, and both of their gene expression levels were significantly upregulated, indicating that these proteins are involved in interactions with insect hormones. Furthermore, there were some unannotated proteins, whose expression patterns were similar after JH induction, such as c8545, c5165, and c5329, but these proteins may be unique to *H. satumaensis* (Table S7-3).

To verify the gene expression profile, 57 genes were randomly selected for quantitative reverse transcription PCR (qRT-PCR) (Table S8). OR225121 was the actin gene in *H. satumaensis* and was used as a reference. Following induction by 20E and JH, the expression of most of the extracellular secretory protein genes increased. To validate their expression levels, we screened extracellular secretory protein sequences from the significantly differentially expressed genes in the 20E and JH treatment groups, concentrating on the genes that were significantly up- and downregulated in both the transcriptome and proteome groups for expression confirmation.

**Fig 7:**
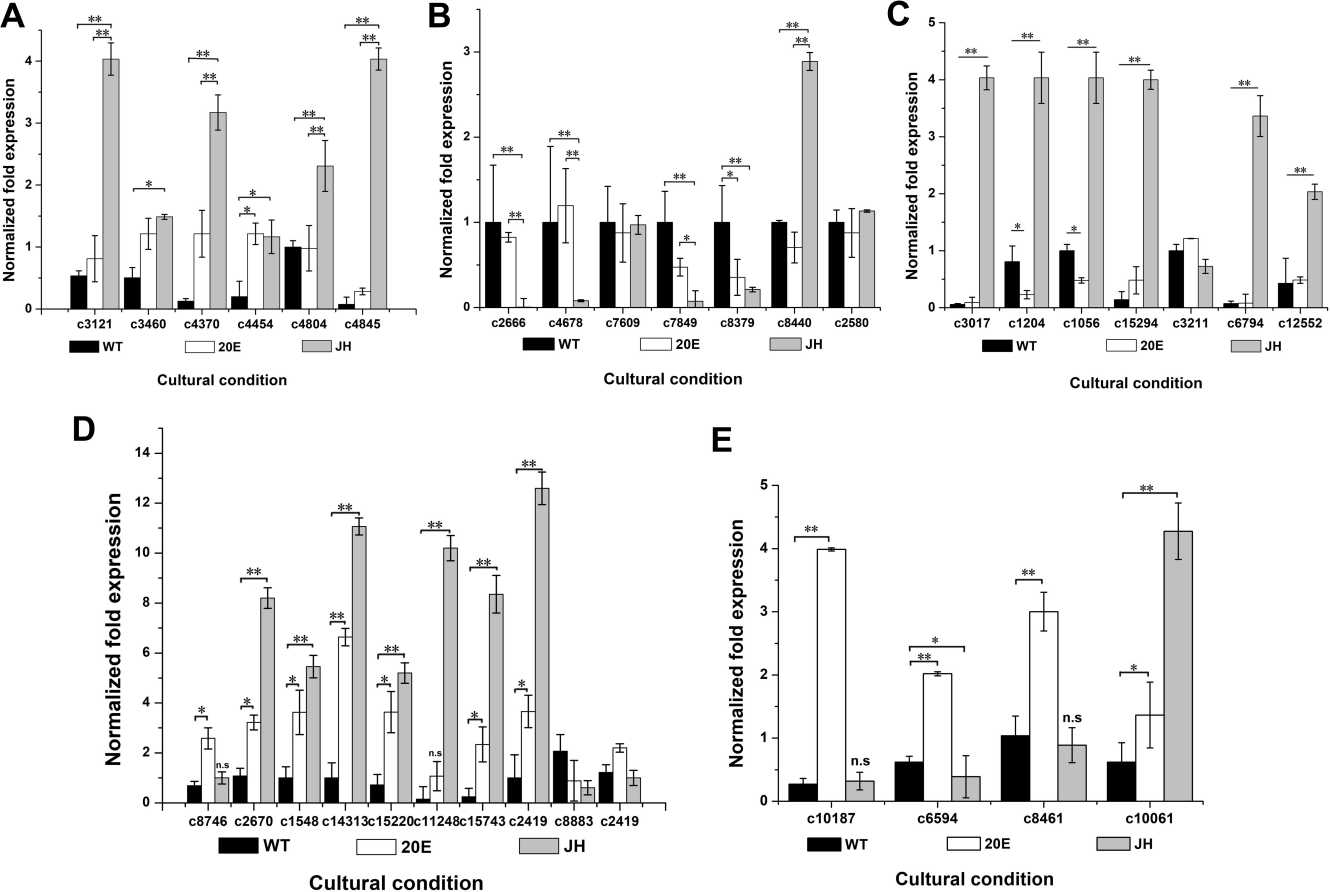
Validation of transcript/protein expression changes under 20E and JH induction conditions by qRT-qPCR. "**" and "*" indicate that the expression levels of two strains are significantly different at *P* < 0.01 and *P* < 0.05, respectively. (A) The representative secreted proteins that are co-upregulated in both groups. (B) The representative secreted proteins that are co-downregulated in the two hormone groups. (C) The seven representative proteins that are exclusively secreted upon JH induction. (D) The ribosomal metabolism-related genes. (E) The genes related to hormone metabolization.

The six secretory proteins c3121 (hypothetical protein), c3460 (subtilisin-like serine protease precursor), c4370 (hypothetical protein), c4454 (hypothetical protein), c4804 (hirsutellin A), and c4845 (hypothetical protein) were co-upregulated and exhibited elevated expression in both treatment groups, particularly in the 20E induction group (Fig. 7A). Additionally, the representative secretory proteins that were co-downregulated were c2666 (hypothetical protein), c4678 (alkaline phosphatase), c7609 (putative inactive purple acid phosphatase 16), c7849 (hypothetical protein), c8379 (secreted protein), c8440 (hypothetical protein), and c2580 (trypsin-like serine protease). Among these proteins, c8440 was strongly induced in response to JH, whereas other genes were downregulated, and their expression patterns were consistent with the transcriptome data (Fig. 7B). Furthermore, among the DEGs in the JH treatment group, some of those genes were predicted to be secreted proteins, with a notable significant difference in expression levels.

Consequently, the expression of these proteins was analyzed because they might be exclusive to the fungus. Seven representative proteins that might be exclusively secreted upon JH induction were identified: c3017 (hypothetical protein), c1204 (uncharacterized protein), c1056 (fibrohexamerin), c15294 (sericin 1), c3211 (fibroin light chain), c6794 (silk gum protein), and c12552 (hypothetical protein) (Fig. 7C). All of these genes, except for c3211, exhibited a significant increase in expression levels when exposed to JH. The expression patterns of these genes were consistent with the transcriptome data.

As the DEGs in the two treatment groups were significantly enriched in the ribosome metabolic pathway, the verification of the expression levels of representative genes in this pathway was conducted. With the exception of c2419 and c8883, all other ribosome coding genes were significantly increased in both the 20E and JH treatment groups, particularly in the JH treatment group (Fig. 7D), which was consistent with the transcriptome data.

To investigate the association between insect hormone metabolism genes and hormones in *H. satumaensis*, qRT-PCR was used to analyze the expression levels of four relevant genes. In the 20E group, the ecdysone inactivation enzyme-encoding genes c10187 (enzyme EGT), c6594 and c8461 (EcKinases), and c10061 (an aldehyde dehydrogenase related to juvenile hormone synthesis) were detected. The three genes encoding ecdysone-inactivating enzymes were activated by ecdysone but not affected by juvenile hormone III. Compared to its upregulation in response to 20E, the expression level of the c10061 gene was also significantly increased (by fourfold) in response to JH induction, suggesting that this gene is involved in the response to both hormones (Fig. 7E). The expression patterns of most of these genes examined using RT-qPCR analysis were in line with the transcriptome data, suggesting that the transcriptome data and DEG analysis were reliable.

## DISCUSSION

Owing to the complexity and variety of components, as well as the presence of multiple stressors in the insect hemocoel, it is challenging to investigate the effects of insect hormones on fungal pathogens under *in vivo* conditions. Previous studies have revealed that the addition of 4–12 mg/L β-ecdysone and juvenile hormone III had a positive effect on the fermentation of *Cordyceps militaris*, particularly by increasing the production of mycelia, intracellular substances, exopolysaccharides, and cordycepin (32, 33). Moreover, the two insect hormones have been shown to alter the metabolism of *C. militaris* and to regulate extracellular polysaccharides, intracellular polysaccharides, and cordycepin (33), demonstrating that insect hormones can affect the secondary metabolism of EPF. The concentration of 20-hydroxyecdysone in insect larvae has been estimated to be 0.5–2.5 ng/g, whereas that in pupae is significantly greater, at 20–100 ng/g (34). The concentration of 20-hydroxyecdysone in the body of fifth-instar silkworms ranged from 200 to 850 ng/g (35, 36). Moreover, concentrations of 640 pg/µL and 25 pg/µL of JH III were detected in the 5th instar larvae of the pea aphid *Acyrthosiphon pisum* and the ant species *Myrmicaria eumenoides*, respectively (37). Our previous experiments were conducted in a liquid medium in which the levels of ecdysone and juvenile hormone were greater than those in fifth-instar silkworm larvae. Therefore, the excessive use of hormones in fungal culture may not accurately represent the effects of hormones on fungi in nature, which might result in hormonal stress, masking the natural response of the fungi.

In this study, we used a concentration of hormone comparable to the level in *B. mori* to induce *H. satumaensis* mycelia. When the concentrations of JH and 20E reached approximately 250 ng/mL and 400 ng/mL, respectively, there was a marked change in fungal morphology, a significant increase in the spore germination rate, and stimulation of colony pigments and spore mucilage (29). These results could mirror a direct interaction between insect hormones and *H. satumaensis*. Transcriptome analysis revealed that the differentially expressed unigenes in the 20E treatment group were significantly enriched in two metabolic pathways: tyrosine and arginine and proline. The DEGs in response to 20E were primarily related to amino acid synthesis and metabolism, whereas those in response to JH were related to the translation, synthesis, and transport of proteins in ribosomes. Moreover, the downregulated DEGs were enriched in aflatoxin biosynthesis (map 00254) in both groups. We analyzed the genes that are enriched in this pathway within this fungus and only three genes were found to be enriched in this pathway: c5227 (*O*-methyltransferase), c7252 (acetyl-CoA carboxylase), and c3407 (aflatoxin biosynthesis ketoreductase nor-1). Due to the lack of a complete homologous pathway for aflatoxin synthesis, the fungus might not produce aflatoxin.

GSEA revealed that the DEGs in the 20E and JH groups were mainly associated with the ribosome pathway and the MAPK signaling pathway, suggesting that *H. satumaensis* activated these two pathways in response to the two insect hormones. To evaluate the genes expressed in the ribosome pathway between the two treatment groups, we identified three core genes, c10957 (60S ribosomal protein L13-A-like protein, RPL-13A), c13202 (40S ribosomal protein S9, RPS-9), and c15746 (60S ribosomal protein L26, RPL-26). These genes are related to the production and manipulation of proteins. We speculate that the increase in secreted protein, upregulated host interaction-related genes, and amino sugar and some amino acid metabolism-related genes might be involved in the ribosome pathway. However, the underlying mechanism is still unclear, as many changes at the mRNA level were not accompanied by changes in the expression levels of their encoded proteins.

In addition, a number of significantly differentially expressed unigenes related to the response to the two hormones were classified into three functional groups: (1) stress response-related genes that are required for the removal of ROS (e.g., glutathione synthetase, c7144), and response to osmotic stress in the hemocoel, such as signaling pathways for G, mTOR, and MAPK proteins (c5464, c4709, c14508) (2); insect hormone metabolism genes, including ecdysteroid UDP-glucosyltransferase (c10187), ecdysteroid-22-kinase (c6594 and c8461), and a key aldehyde dehydrogenase (c10061) in the juvenile hormone synthesis pathway; and (3) secretory proteins homologous to those found in the host *B. mori*, such as fibrohexamerin, fericin 1, metalloprotease 1 protein, and silk gum protein, which are related to hormone response. In accordance with prior results, there are signaling pathways for G and MAPK proteins that commence with the interaction between a receptor and a ligand (owing to environmental stress), which acts as an activation/deactivation switch of the signaling cascade (38–40).

To compare the mucilage protein profiles of spores induced by ecdysone and juvenile-maintaining hormone, a label-free quantitative proteomics approach was used to identify the secretory proteins (probably present in spore mucilage) expressed in response to the two hormones compared to those in the control treatment. The 20E treatment resulted in 103 differentially expressed proteins compared to those in the control group, while the JH treatment resulted in 224 differentially expressed proteins (Table 3). There were 79 and 7 proteins that were specifically expressed in the 20E and JH treatment groups, respectively (Fig. 5A). GO function and KEGG pathway analyses revealed that *H. satumaensis* in response to 20E mainly involved lipid metabolism and amino carbohydrate metabolism. The proteins differentially expressed in the JH treatment group were primarily related to glutathione metabolism (ko00480); phenylalanine, tyrosine, and tryptophan biosynthesis (ko00400); and glycine, serine, and threonine metabolism (ko00260). These findings were consistent with the transcriptome data suggesting that the amino acid metabolic pathway is involved in the response to insect hormones.

Insect hormones can affect the colonization of fungal pathogens within insects. It has been demonstrated that, during the early stages of fungal infection, insects can manage the molting process by increasing the concentration of ecdysone to reduce the binding of the fungi to their outer surface, thereby preventing fungal infection (40). Upon infection of *Galleria mellonella* larvae by *B. bassiana*, the level of ecdysone (20E) detected in the larval hemolymph gradually increased. In addition, when the concentration of 20E in insects is reduced by inactivation enzymes, the mortality of hosts infected by *B. bassiana* significantly increases (20). Our previous study showed that conidial germination slightly decreased, while sporulation initially decreased and then increased with increasing hormone concentration in media (29). Our findings suggested that 20E may serve as a stressor, hindering spore germination while promoting the production of conidia and pigments.

20E is also involved in the regulation of the immune system (41–43). As an evolutionary strategy, some insect fungal pathogens have evolved inactivating enzymes (ecdysteroid-22-oxidase, MrE22O) that target ecdysone and can effectively reduce the concentration of this hormone and weaken the immune response of insects. MrE22O is an ecdysone-inactivating enzyme from *Metarhizium rileyi*. It specifically oxidizes the hydroxyl group on ecdysone C22, resulting in the formation of a carbonyl group and the inactivation of ecdysone (44, 45). Overexpression of this enzyme in *B. bassiana* results in increased virulence and a weakened host immune response (20). Our results also showed that some specific ecdysone-inactivating enzymes (ecdysteroid UDP-glucosyltransferase, EGT, and EcKinase) are present in *H. satumaensis*, suggesting that the fungal pathogen has evolved multiple ecdysone-inactivating enzymes to disrupt the signal of insect hormones.

Our previous study indicated that the hydrophobicity and virulence of spores are significantly impacted by the presence of a mucilage layer on *H. satumaensis* conidia. Once the mucilage is removed, the surface of the spores becomes hydrophilic, and the virulence of *H. satumaensis* to *Galleria mellonella* and *Plutella xylostella* decreases by 6.7% and 17.66%, respectively (46–48). This research also revealed that enzymes associated with the breakdown of the host cuticle wall are present in mucilage, including insect epidermal degradation enzymes, proteases, chitinases, and lipases, suggesting that mucilage plays a role in the process of host cuticle penetration. Our data revealed a total of 1,016 mucilage proteins, 474 of which overlapped with the transcriptome. Among these proteins, seven were related to surface adhesion, including annexin, mucin, secretory protein, and secretion pathway protein Sls2/Rcy1. Further identification of mucilage proteins revealed nine proteins that were specifically expressed and seemed to participate in defense mechanisms. These proteins include bactericidal permeability increasing proteins, pH response regulatory proteins, ABC family transporters, heat shock proteins, multidrug resistance proteins, multidrug resistance transporters, penicillin-binding proteins, catalase, and peroxidase.

Combining the transcriptomic and proteomic data sets revealed a phenomenon: the expression of a total of 1,681 mRNAs and 3,620 mRNAs changed in response to 20E and JH, respectively, while the expression of 103 proteins and 224 proteins in the mucilage did so. Moreover, 80 and 114 DEGs encoding extracellular secretory proteins were identified from the transcriptomic data of the 20E and JH groups, respectively. However, only 25 and 30 candidates displayed significant changes in gene expression and protein abundance in both the transcriptome and proteome, respectively, in the 20E and JH groups. In terms of consistency, 7 and 10 genes showed consistent expression of both mRNAs and corresponding proteins in the 20E and JH treatment groups, respectively (Table 3). Most of the alterations in mRNA expression did not correspond to changes in mucilage protein levels. Furthermore, *H. satumaensis* primarily exhibited an upregulation of ecdysone-inactivating enzymes in response to 20E, whereas an increase in secretory proteins homologous to the host was observed in response to JH. This difference may be caused by differences in the functions of the two insect hormones. Insect development is cooperatively orchestrated by 20E and JH. Research has revealed that ecdysone and JH can mutually repress their biosynthesis by inhibiting the transcription of enzymes involved in biosynthetic pathways (49, 50), and the JH signaling pathway negatively regulates the transcription of genes involved in ecdysone signaling (51, 52). Undoubtedly, 20E can regulate the strength of the insect immune reaction, and fungi have evolved inactivated enzymes accordingly. Although the connection between JH and insect immunity remains uncertain, it is closely linked with 20E in insects and is likely to impact fungal colonization. Based on the transcriptome and proteome data, we concluded that the fungus responds to hormone signaling factors by activating MAPK and mTOR, leading to a cascade of changes in virulence, cell-cell signaling, fungus-insect interactions, and response to damage-associated molecular patterns, as well as other transcriptional changes.

In conclusion, upon exposure to the insect ecdysone (20E) and juvenile hormone III (JH), *H. satumaensis* increases the production of many secreted proteins, insect hormone metabolism proteins, and lectin-like proteins. These findings demonstrate that hormones might serve as signaling factors for EPF during colonization in insects. To survive the physiological stress caused by hormones, EPF have evolved specialized adaptive strategies in which ecdysone-inactivating enzymes are produced to overcome hormone-mediated stress or immune responses, thus expanding the range of available strategies. For example, by overexpressing inactivating enzymes in entomopathogenic fungi, it is feasible to create improved engineered strains that have faster knockdown effects on host insects and better environmental adaptation.

## MATERIALS AND METHODS

### Strains and growth conditions

The strain of *Hirsutella satumaensis*, known as GZUIER-Hir201012JC, was obtained from Suiyang County, Guizhou Province, and maintained at the Institute of Fungal Resources of Guizhou University. The culture was maintained on potato dextrose agar (PDA). Conidia were obtained by growth at 22°C for 10 days under 12 h light/12 h dark conditions.

### Induction culture of two insect hormones

Following the method of Yang et al. (29) with slight improvements, the conidia were harvested and used to prepare a suspension at a concentration of 1 × 10^6^ cells/mL in 0.05% Tween 80. Two microliters of the suspension were inoculated on PDA covered with cellophane and cultured at 22°C for 7 days under 12 h light/12 h dark conditions. The cellophane was then transferred to fresh PDA plates containing two insect hormones, namely, 20E at a concentration of 400 ng/mL and JH III at 250 ng/mL, for a further 5 days. Mycelia were transferred to the new PDA plates without insect hormones as control groups. The mycelia were immediately cryopreserved in liquid nitrogen until RNA extraction. Three biological replicates were prepared for mRNA-Seq in both control and treatment groups.

### RNA extraction and *de novo* assembly of *H. satumaensis* transcriptome

Total RNA was extracted from the above mycelia using TRIzol Reagent according to the manufacturer’s instructions (Invitrogen, Carlsbad, CA, USA), and the genomic DNA was removed using DNase I (TaKaRa). The integrity and purity of the total RNA quality were determined by 2100 Bioanalyser (Agilent Technologies, Inc., Santa Clara CA, USA) and quantified using the ND-2000 (NanoDrop Thermo Scientific, Wilmington, DE, USA). Only high-quality RNA samples (OD_260/280_ = 1.8–2.2, OD_260/230_ ≥ 2.0, RIN ≥ 8.0, 28S:18S ≥ 1.0, >1 µg) were used to construct sequencing library (Fig. S14). RNA purification, reverse transcription, library construction, and sequencing were performed at the Shanghai Majorbio Bio-pharm Biotechnology Co., Ltd. (Shanghai, China) according to the manufacturer instructions (Illumina, San Diego, CA). The nine RNAseq libraries were sequenced in a single lane on an Illumina Hiseq xten/NovaSeq 6000 sequencer (Illumina, San Diego, CA) for 2 × 150 bp paired-end reads.

The Control, +20E and +JH groups, with three biological replicates for each sample, yielded a total of 49,127,926 to 61,846,398 raw reads. After filtering and trimming the data, the number of high-quality clean reads ranged from 47,870,786 to 60,459,720 bp with a Q20 percentage greater than 97% and a GC percentage ranging from 61.44% to 62.29% (Table S9). Raw paired-end reads were trimmed and quality controlled using SeqPrep (https://github.com/jstjohn/SeqPrep) and Sickle (https://github.com/najoshi/sickle) with default parameters. Clean data from the samples (*H. satumaensis*) were then used to perform *de novo* assembly using Trinity (http://trinityrnaseq.sourceforge.net/) (53). All assembled transcripts were searched against the NR, COG, and KEGG databases using BLASTX to identify the proteins with the highest sequence similarity to the given transcripts to retrieve their function annotations, with a typical cutoff E-value of less than 1.0 × 10^−5^. The BLAST2GO program (http://www.blast2go.com/b2ghome) was used to obtain GO annotations of uniquely assembled transcripts to describe biological processes, molecular functions, and cellular components (54). Metabolic pathway analysis was performed using the Kyoto Encyclopedia of Genes and Genomes (KEGG; http://www.genome.jp/kegg/) (55).

### Analysis of differentially expressed genes

To identify differential expression genes (DEGs) between two different samples, the expression level of each transcript was calculated using the transcripts per million reads (TPM) method. RSEM (http://deweylab.biostat.wisc.edu/rsem/) was used to quantify gene abundances (31). Essentially, differential expression analysis was performed using the DESeq2 with *Q*-value ≤ 0.05; DEGs with |log2FC| > 1 and *Q*-value ≤0.05 (DESeq2) were considered significantly differentially expressed genes (56). In addition, functional enrichment analysis, including GO and KEGG, was performed to identify which DEGs were significantly enriched in GO terms and metabolic pathways with Bonferroni-corrected *P*-value ≤ 0.05 compared to the whole transcriptome background. GO functional enrichment and KEGG pathway analyses were carried out by Goatools (https://github.com/tanghaibao/Goatools) and KOBAS (http://bioinfo.org/kobas/), respectively (57). Hierarchical clustering analysis was performed using the fastcluster package.

### Extraction of total proteins from conidial mucilage and label-free quantitative proteomic analysis

The fungal culture was prepared according to the approach described for the transcriptome analysis. The protein extraction method was described by Duan et al. (30). Briefly, the mature conidia were collected and processed using a phosphate buffer (pH 7.2) to prepare a sporulation suspension at a density of 5 × 10^7^ cells/mL. Each sample was supplemented with dithiothreitol (DTT), a reducing agent until the concentration reached 2 mmol/L and the total volume was up to 10 mL. The extraction program was performed at 4°C and 100 rpm/min for 24 h. The mixture was then centrifuged at 4°C and 7,104 *g* for 10 min. The supernatant was collected, and 20 mg/mL of sodium deoxycholate was added to give a final concentration of 300 µg/mL. After overnight precipitation on ice, the mixture was centrifuged at 4°C and 11,000 *g* for 10 min. The protein sediment was transferred to a fume hood to evaporate the residual acetone and was finally stored at −80°C.

The protein, 250 µg per sample, was digested using the FASP procedure as reported by Wisniewski et al. (58) and desalted using C18 cartridges, followed by concentration via vacuum centrifugation and reconstitution in 40 µL of a solution containing 0.1% (vol/vol) trifluoroacetic acid. MS experiments were performed on a Q Exactive mass spectrometer coupled to a Vanquish Neo UHPLC. The peptide sample of five micrograms was loaded onto a C18 reversed-phase column pre-equilibrated with buffer A consisting of 2% acetonitrile and 0.1% formic acid. The separation was performed using a linear gradient of buffer B (80% acetonitrile and 0.1% formic acid) at a flow rate of 250 nL/min, controlled by IntelliFlow technology, for 120 min. To obtain MS data, a data-dependent top10 technique was used to select the most abundant precursor ions for HCD fragmentation from the survey scan (300–1,800 *m*/*z*). The determination of the target value depends on the implementation of a predictive automatic gain control. Survey scans were acquired at a resolution of 70,000 at *m*/*z* 200, and the resolution for HCD spectra was set to 17,500 at *m*/*z* 200. Triplicate MS experiments were performed for each sample to ensure the validity and accuracy of the analysis. MS data were analyzed using MaxQuant software version 1.3.0.5 and searched against the UniProtKB database. To facilitate database searching, cysteine modification by carbamidomethylation was considered a fixed modification, while protein N-terminal acetylation and methionine oxidation were considered variable modifications. The global false discovery rate (FDR) cutoff for peptide and protein identification was set at 0.01. Label-free quantification was performed using MaxQuant. Protein abundance was calculated from the normalized spectral protein intensity (LFQ intensity).

### Analysis of differentially expressed proteins

GO annotations of the *H. satumaensis* proteome were obtained from the UniProt-GOA database (www. http://www.ebi.ac.uk/GOA/). InterProScan (http://www.ebi.ac.uk/interpro/) was used to annotate protein GO functions based on the protein sequence alignment method (59). The enrichment levels of all proteins identified from *H. satumaensis* were compared with those from the databases based on their respective GO annotation categories using a two-tailed Fisher’s exact test. GO terms showing significant enrichment were identified based on a corrected *P*-value of less than 0.05.

### Correlation analysis of transcriptomic and proteomic data

To understand the correlation between transcriptomic and proteomic data in different hormone-induced groups, the genes and proteins that co-occurred in both omics data were screened. According to the relative difference multiples between the transcriptome and the proteome, the default screening threshold was set to two times that of the proteins and the transcript group. After log2 transformation, GraphPad Prism 8 (v9.4.0) was used to generate a nine-quadrant map of transcripts and proteins of differential expression.

### qRT-PCR analysis

Validation of mRNA-Seq data was performed for both transcriptome and proteome coding genes using quantitative reverse transcription PCR (qRT-PCR). Total RNA was isolated using the RNeasy Plant Mini Kit (Qiagen, Maryland, USA), and DNA contamination in the RNA samples was removed using RQ1 RNase-free DNase (Promega, Madison, USA). Two micrograms of total RNA were reverse transcribed using an oligo (dT)-primed cDNA synthesis kit (MBI Fermentas, Burlington, CA). Real-time PCR analyses were performed using a quantitative real-time PCR kit (Bio-Rad, USA) with intron-F and intron-R primers (Table S8) designed for validation genes. Forty-seven qPCR primers were used in qRT-PCR analyses to validate the RNA-seq results. Primers were designed based on the coding regions of the contigs using the online program Primer-BLAST (https://www.ncbi.nlm.nih.gov/tools/primer-blast). The sequences of all primers used in this study are listed in Table S8. The expression level of the reference gene, actin, was determined using the actin-F and actin-R primers. These levels were then used to normalize the transcript levels of the gene using CFX Manager software (Bio-Rad, Hercules, USA), as described by He et al. (60).

### Statistics

Statistical significance was determined using GraphPad Prism 7; Student’s unpaired two-tailed *t*-test, one-way ANOVA, or two-way ANOVA was conducted according to the test requirements. *P < 0.05, **P < 0.01, and ***P < 0.001 were considered significant. The number of replicates and repetitions for individual experiments and statistical tests are indicated in the legends.

## Data Availability

Data reported here have been deposited in the GenBank database. RNA-seq data were submitted to the Sequence Read Archive (SRA) database at NCBI under the following accession numbers: PRJNA984006 for the control group; PRJNA985362 for the induction culture of *H. satumaensis* with 20E; and PRJNA985363 for the induction culture of *H. satumaensis* with JH III. The mass spectrometry proteomics data were deposited at the ProteomeXchange Consortium (http://proteomecentral.proteomexchange.org) via the iProX partner repository (61, 62) with the data set identifier PXD043411 (https://www.iprox.cn/page/project.html?id=IPX0006651000).

## SUPPLEMENTAL MATERIAL

Supplemental Figures msystems.00166-24-s0001.pdf Fig. S1-S14.

Supplemental Tables msystems.00166-24-s0002.xlsx Tables S1-S9.

ASM does not own the copyrights to Supplemental Material that may be linked to, or accessed through, an article. The authors have granted ASM a non-exclusive, world-wide license to publish the Supplemental Material files. Please contact the corresponding author directly for reuse.
