# Supplementary material for: Transcriptome and proteome analyses reveal genes and signaling pathways involved in the response to two insect hormones in the insect-fungal pathogen Hirsutella satumaensis
Source: mSystems. 2024 Jul 10;9(8):e00166-24. doi: 10.1128/msystems.00166-24 (PMC11334460; doi:10.1128/msystems.00166-24)
Supplement: Supplemental Figures — Fig. S1-S14. [file msystems.00166-24-s0001.pdf]

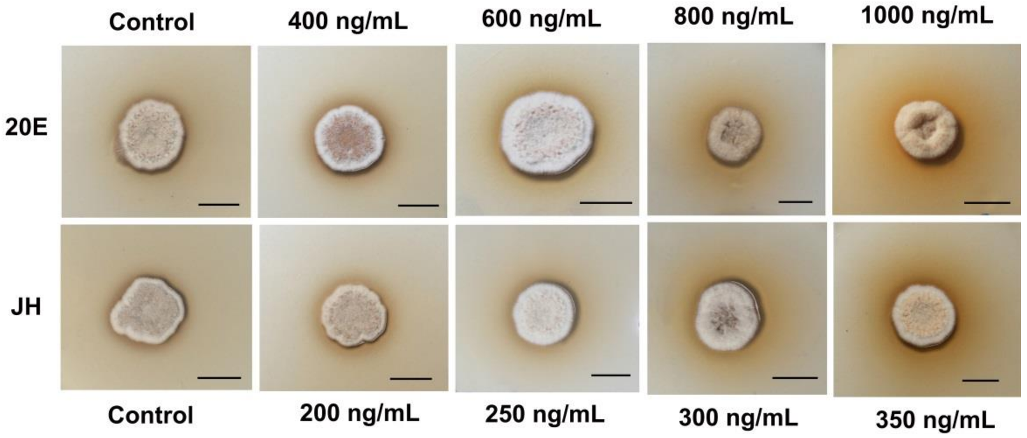

FIG S1 Effects of two insect hormones on the morphological characteristics of *Hirsutella satumaensis* colonies. Bar=1 cm. 20E, ecdysone; JH, juvenile hormone III (same below)

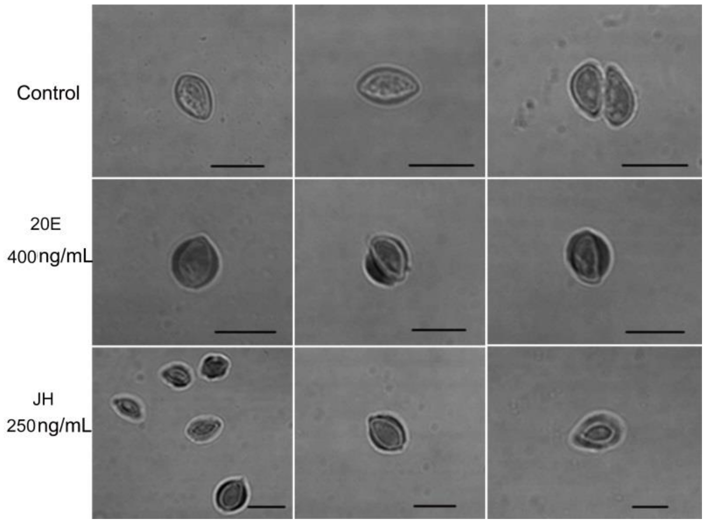

FIG S2 Effects of two hormones on the thickness of conidial mucilage of *H. satumaensis*. Bar=5 μm

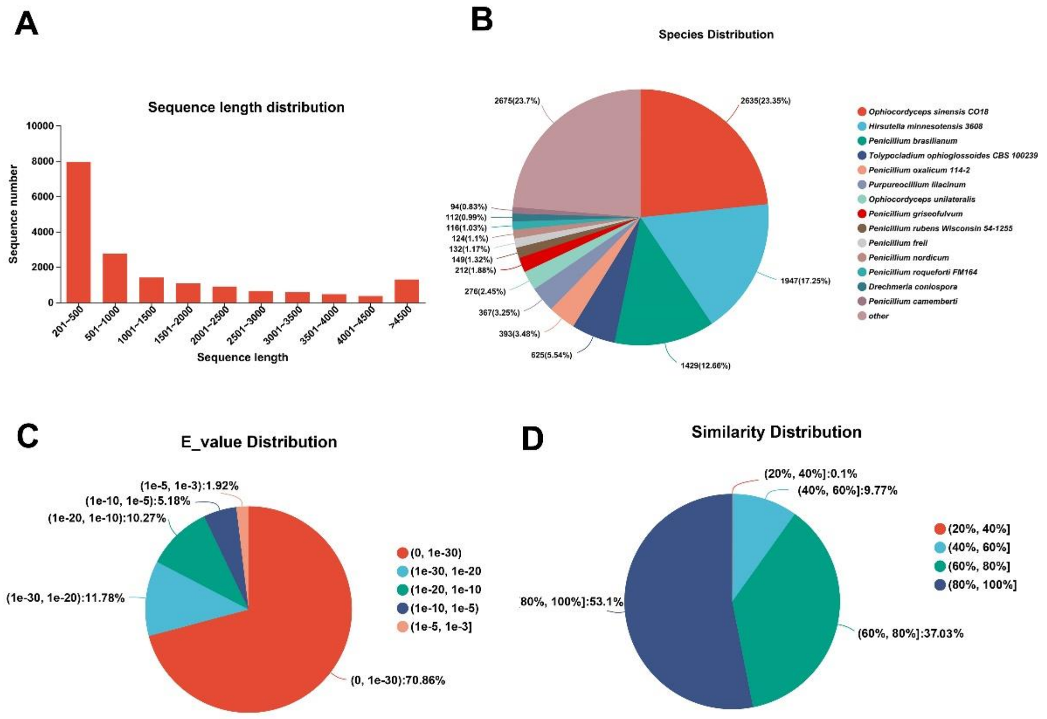

FIG S3 Supporting information of the identified *H. satumaensis* unigenes.
The size distribution (A), species distribution (B), E-value distribution (C), similarity distribution (D) of *H. satumaensis* unigenes matched to the NR database.

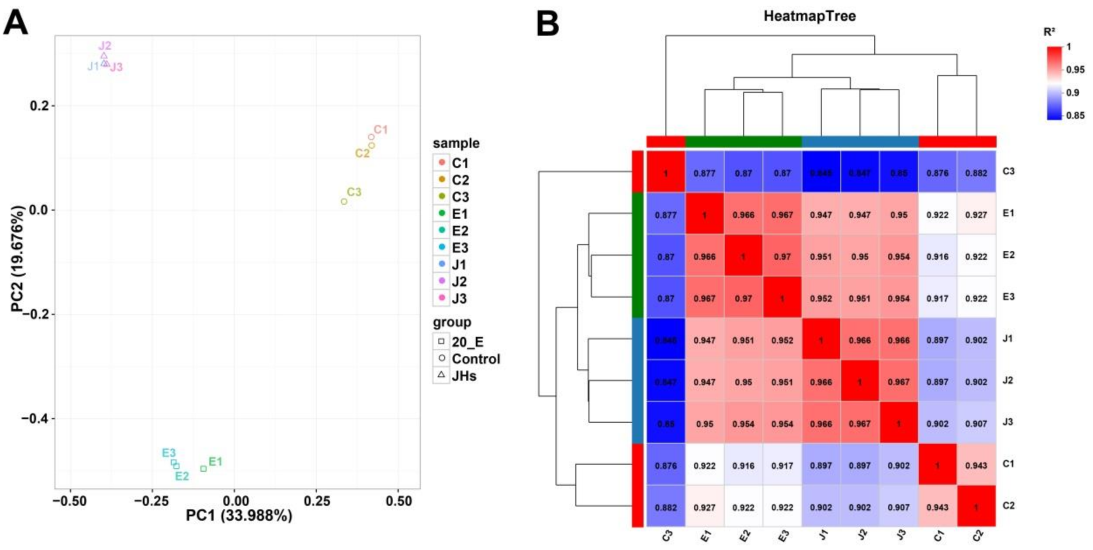

FIG S4 Intersample principal component analysis
A, PCA analysis diagram of samples; B, heatmap of correlation coefficients among samples. Note: After dimensionality reduction analysis, samples have relative coordinate points on principal components. The distance of each sample point represents the distance between samples. The closer the distance is, the more similar between samples are. The horizontal axis represents the cumulative variance contribution of Component 1 (PC1) in the two-dimensional plot to the differentiated samples, and the vertical axis represents the cumulative variance contribution of Principal Component 2 (PC2) in the two-dimensional plot to the differentiated samples.

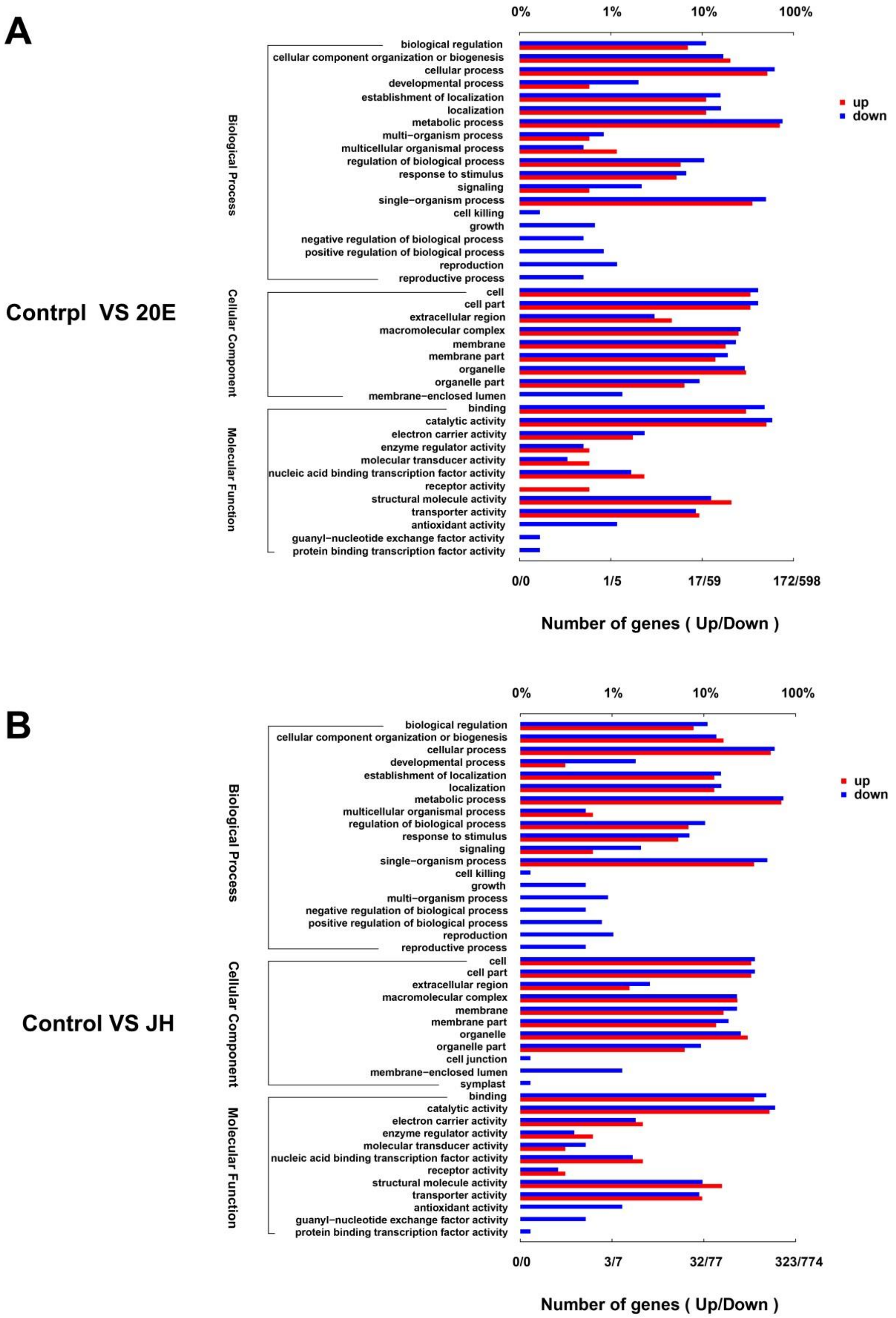

FIG S5 Go class of DEGs associated with *H. satumaensis* under 20E and stress. Note: Unigenes upregulated more than 2-fold and downregulated less than 0.5-fold were subjected to analysis by BLAST2GO. DEGs were identified as being involved in the three GO categories: (a) biological process, (b) cellular component, and (c) molecular function.

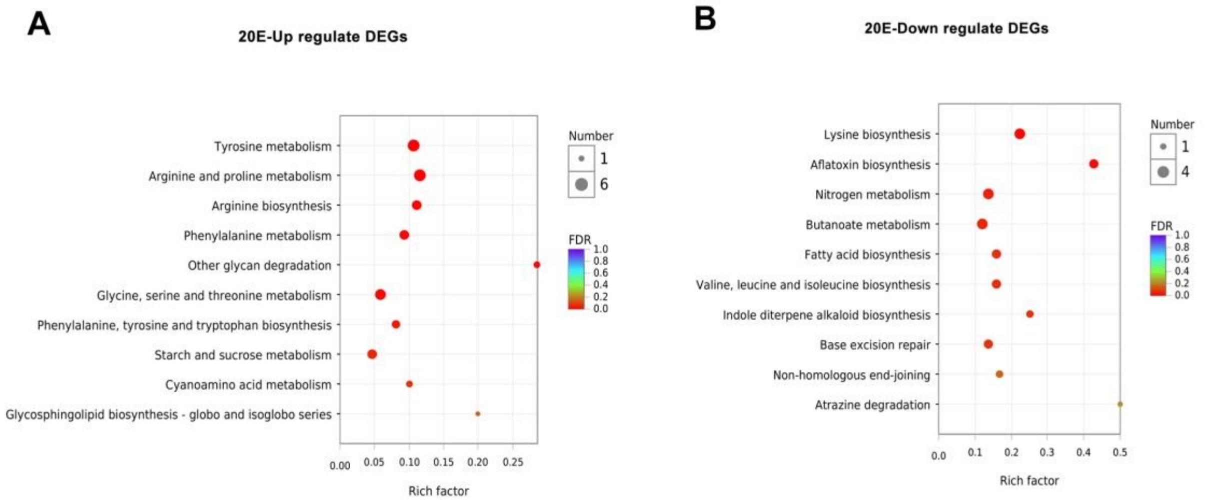

FIG S6 KEGG enrichment analysis of significantly up- and downregulated genes of *H. satumaensis* under 20E stress. Note: The vertical axis represents the pathway name, and the horizontal axis represents the ratio of the number of transcripts/unigenes enriched in the pathway to the number of annotated transcripts/unigenes (background number). The larger the Rich factor is, the greater the degree of enrichment is. The size of the dot indicates the number of transcripts/unigenes in the pathway.

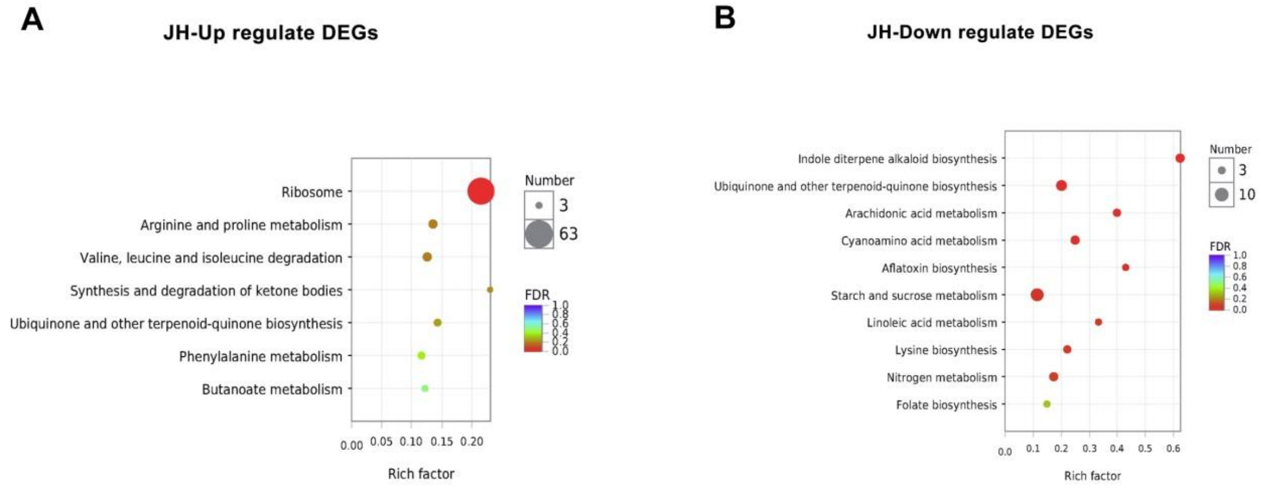

FIG S7 KEGG enrichment analysis of significantly up- and downregulated genes of *H. satumaensis* under JH stress. Note: The vertical axis represents the pathway name, and the horizontal axis represents the ratio of the number of transcripts/unigenes enriched in the pathway to the number of annotated transcripts/unigenes (background number). The larger the Rich factor is, the greater the degree of enrichment is. The size of the dot indicates the number of transcripts/unigenes in the pathway.

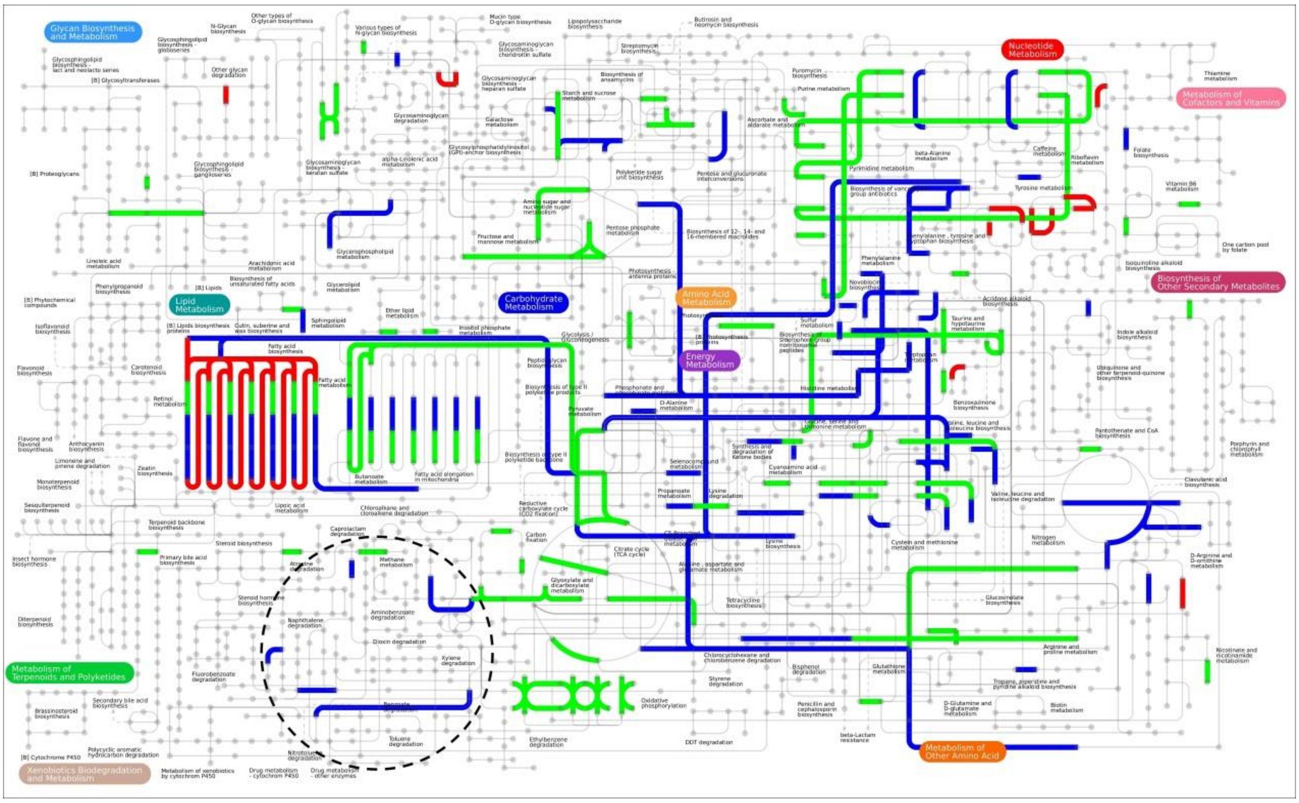

FIG S8 The visualized metabolic pathway enriched by significantly differentially expressed genes of *H. satumaensis* under the 20E and JH induction. Note: Red represents the gene annotation pathway in the gene set of 20E; light green for JH; the blue color represents the common annotated pathway of genes in the two gene sets. Map: Metabolic pathways.

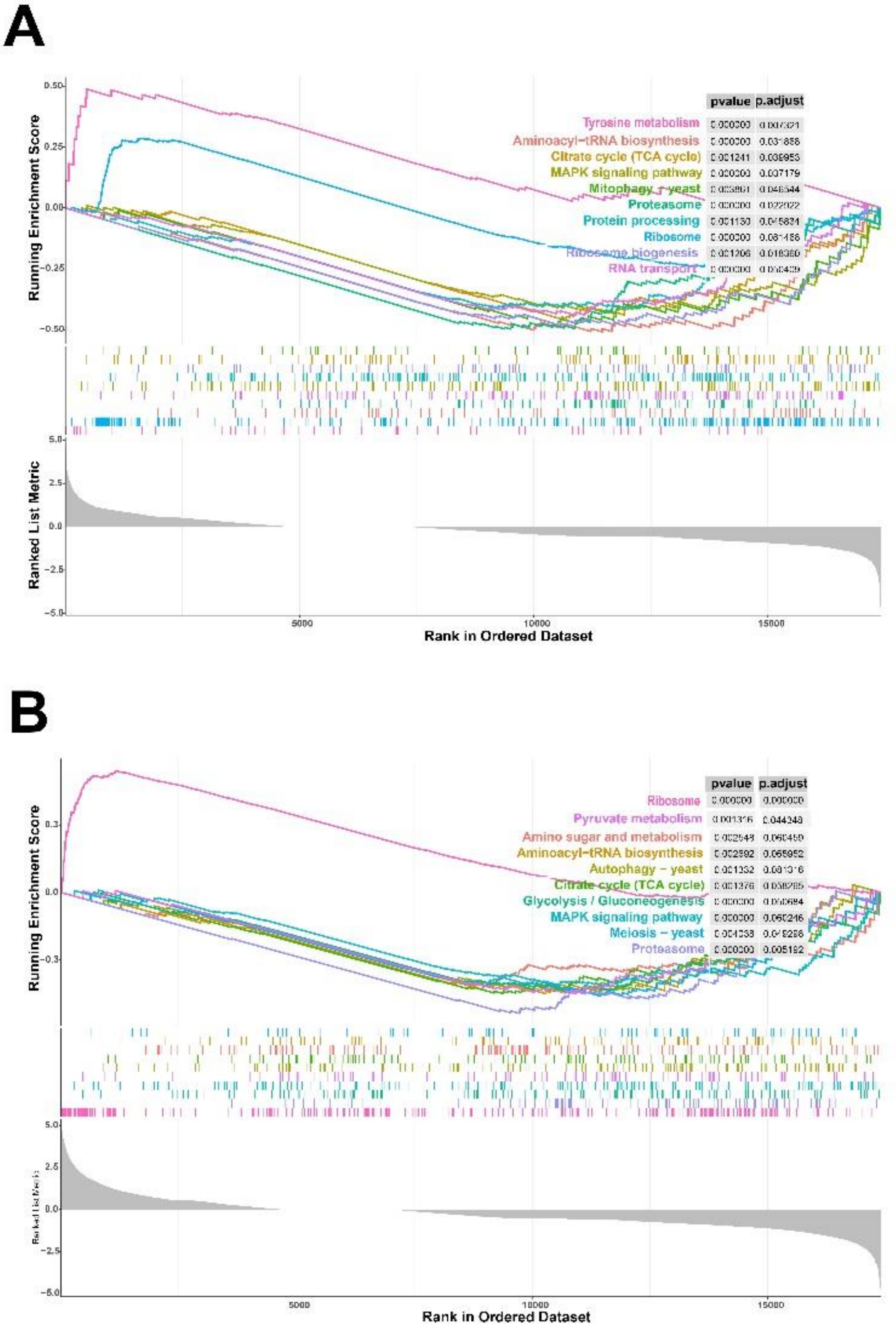

FIG S9 Gene set enrichment analysis (GSEA) revealed an increased enrichment of metabolism pathways associated with the two insect hormones in *H. satumaensis*. GSEA shows the top 10 enriched pathways using enrichment plots induced by 20E (A) and JH (B), respectively.

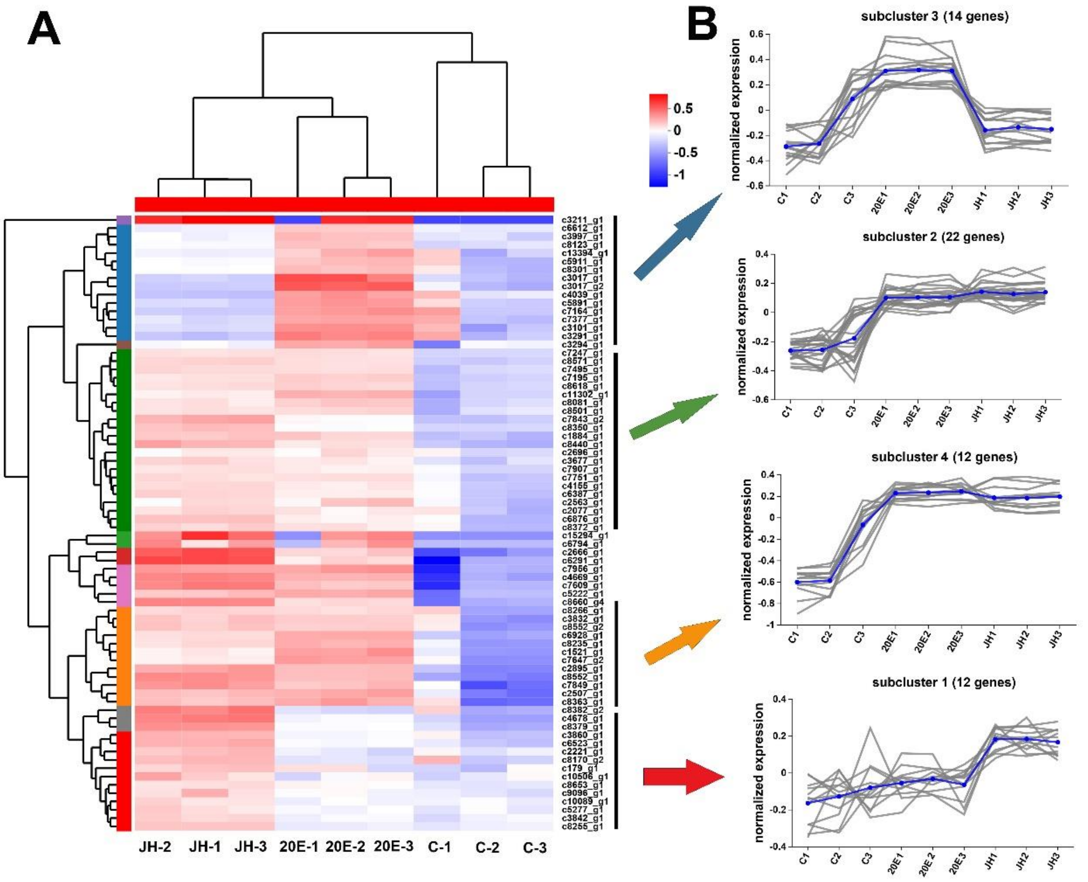

FIG S10 Analysis of expression patterns of differentially expressed secreted proteins from transcriptome data induced by 20E and JH. A, Heat map visualizing the secreted proteins. B, Clustering analysis of the secreted proteins resulted in four clusters: all upregulated by the two hormones (subclusters 2 and 4), upregulated by 20E (subcluster 3), and expressed upon JH treatment (subcluster 1). Values in y-axis are Z-scores.

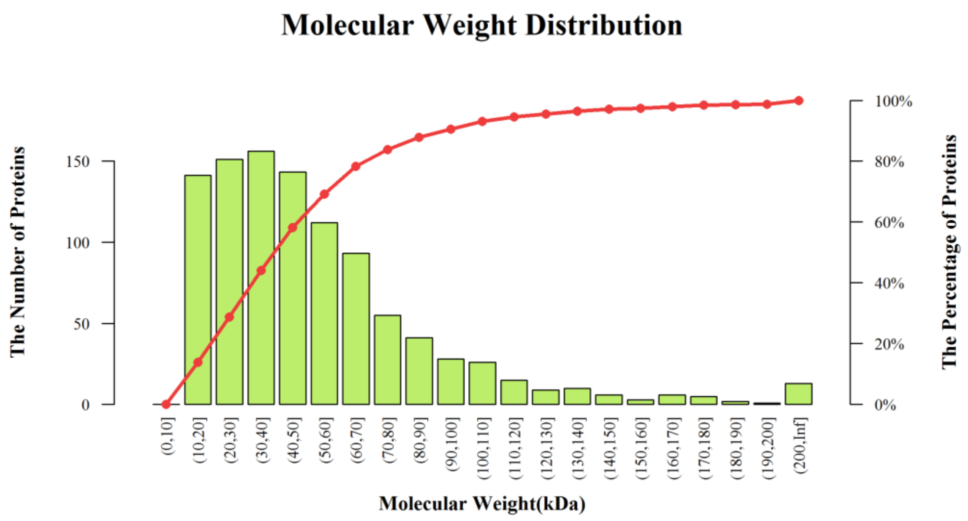

FIG S11 Molecular weight distribution of the conidial mucilage proteins of *H. satumaensis.*

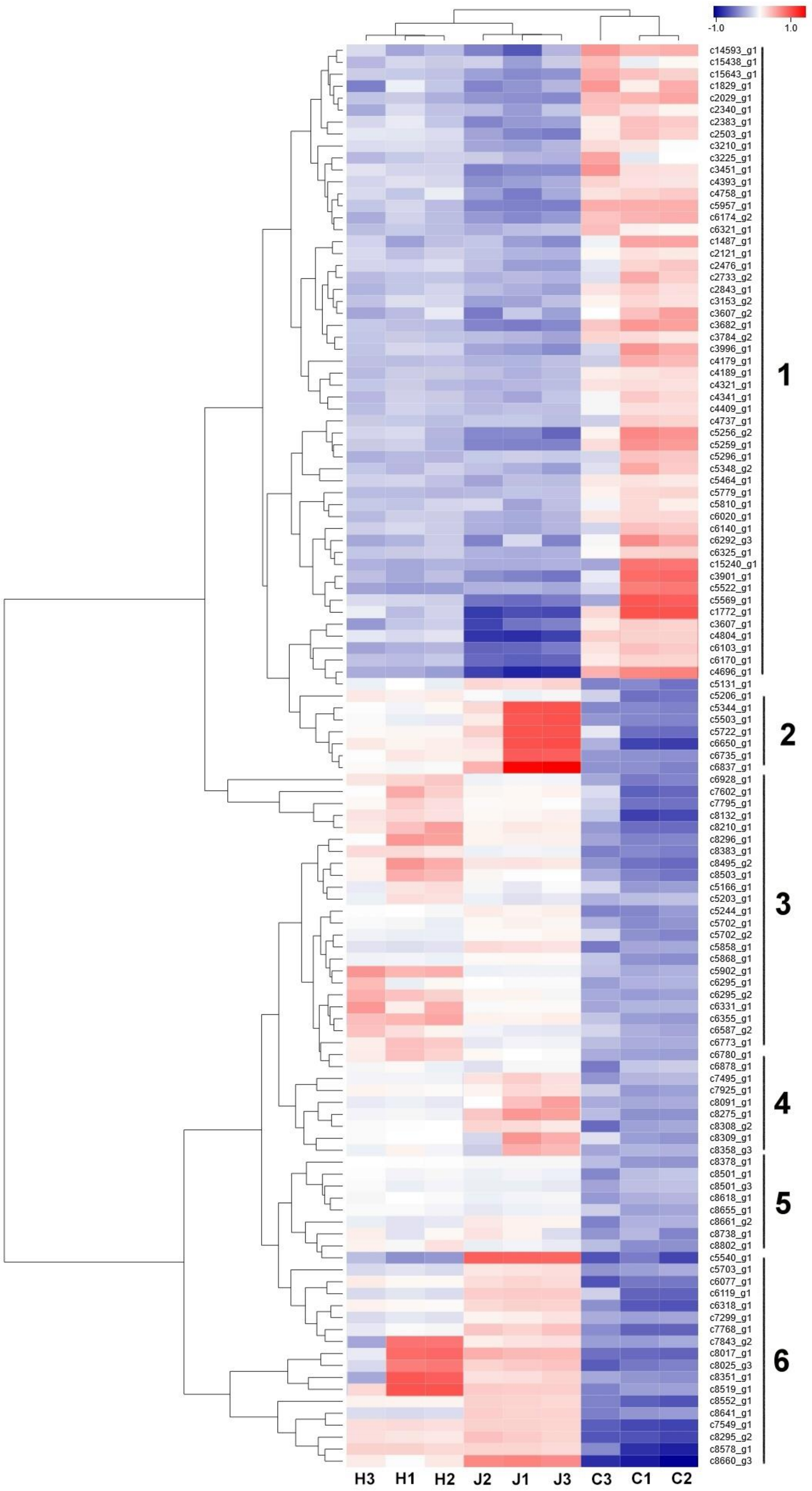

FIG S12 Hierarchical clustering of co-identified mucilage proteins in control, 20E and JH. Dendrogram of experimental group clustering is shown on the top and of individual proteins are shown on the left with a relative expression values displayed as a heat map. C, control; H, 20E; J, JH.

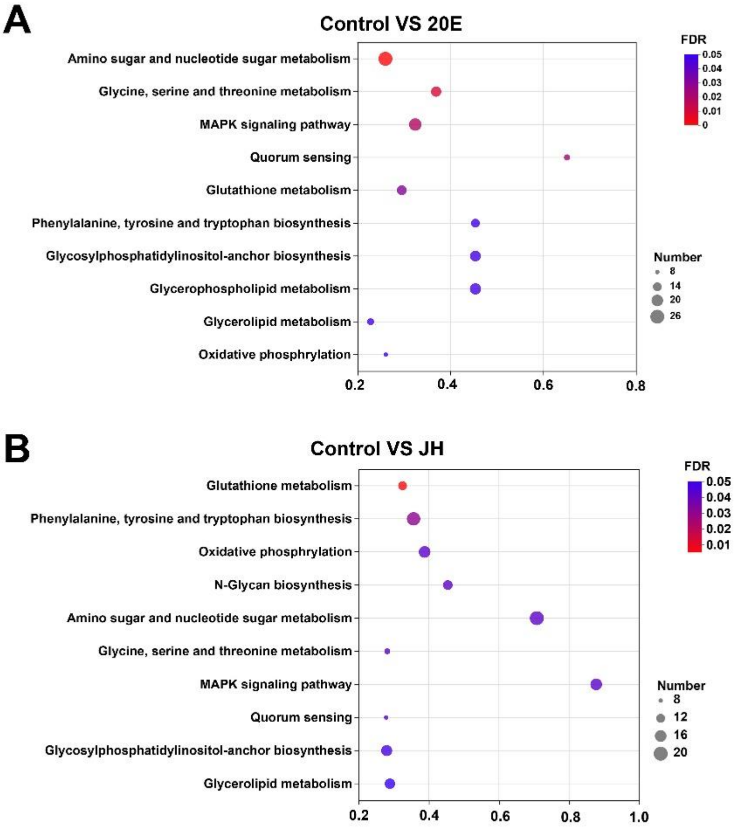

FIG S13. KEGG enrichment analysis of differentially expressed proteins (DEPs) induced by 20E and JH. If the P value is smaller (P<0.05), the item's corresponding enrichment is more significant.

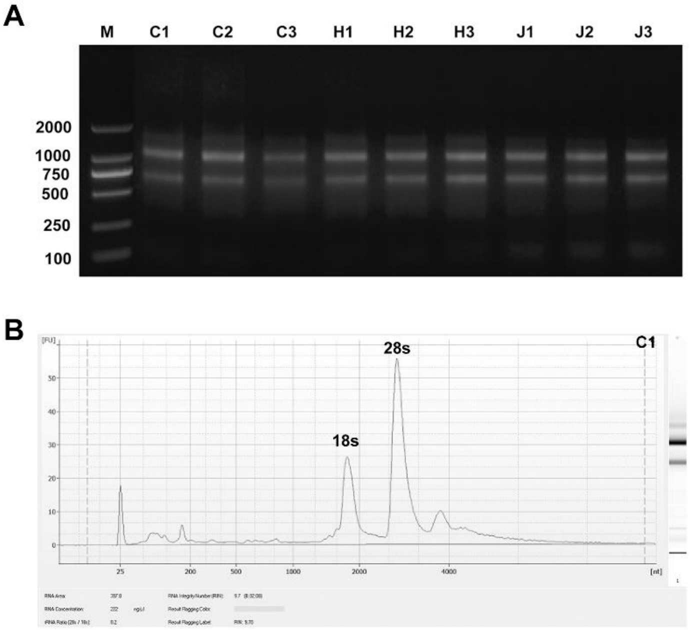

Fig S14 Total RNA quality detection results of all samples

A, total RNA of 9 samples was detected by electrophoresis; B, Agilent 2100 detection result of C1 sample. C represents the control group, H represents the 20E-treated group, and J represents the JH III-treated group.
